# Emergence of slip-ideal-slip behavior in tip-links serve as force filters of sound in hearing

**DOI:** 10.1038/s41467-024-45423-8

**Published:** 2024-02-21

**Authors:** Nisha Arora, Jagadish P. Hazra, Sandip Roy, Gaurav K. Bhati, Sarika Gupta, K. P. Yogendran, Abhishek Chaudhuri, Amin Sagar, Sabyasachi Rakshit

**Affiliations:** 1 Department of Chemical Sciences, Indian Institute of Science Education and Research Mohali, Punjab, India; 2 Department of Physical Sciences, Indian Institute of Science Education and Research Mohali, Punjab, India; 3 National Institute of Immunology, New Delhi, India; 4 Centre de Biologie Structurale, INSERM, CNRS, Université de Montpellier, Montpellier, France

**Keywords:** Single-molecule biophysics, Molecular conformation

## Abstract

Tip-links in the inner ear convey force from sound and trigger mechanotransduction. Here, we present evidence that tip-links (collectively as heterotetrameric complexes of cadherins) function as force filters during mechanotransduction. Our force-clamp experiments reveal that the tip-link complexes show slip-ideal-slip bond dynamics. At low forces, the lifetime of the tip-link *complex* drops monotonically, indicating slip-bond dynamics. The ideal bond, rare in nature, is seen in an intermediate force regime where the survival of the *complex* remains constant over a wide range. At large forces, tip-links follow a slip bond and dissociate entirely to cut-off force transmission. In contrast, the *individual* tip-links (heterodimers) display slip-catch-slip bonds to the applied forces. While with a phenotypic mutant, we showed the importance of the slip-catch-slip bonds in uninterrupted hearing, our coarse-grained Langevin dynamics simulations demonstrated that the slip-ideal-slip bonds emerge as a collective feature from the slip-catch-slip bonds of *individual* tip-links.

## Abstract (web summary)

In this work, the authors report that tetrameric tip-link complex, pivotal for force transmission, forms slip-ideal-slip bonds under tension, serving as efficient force-filters in the auditory process.

## Introduction

Sound as stimuli induces oscillations in the inner ear fluid and deflects the top of the stereocilia cluster that are protruding from the apical end of hair cells^1^. The tips of two adjacent stereocilia in a row of the hair-bundle are connected by a filamentous cadherin complex known as tip-links. The deflection of stereocilia inflicts tension on tip-links^2^. Tip-links convey the tension to mechanoelectrical transduction (MET) channels for response. The intensity of the input sound governs the number of transduction channels opening^3^. Thus, tip-links were conceptualized as gating-springs^4^ in the mechano-transduction process of hearing that sense tension from various auditory inputs and trigger MET channel opening beyond a threshold.

**Fig. 1:**
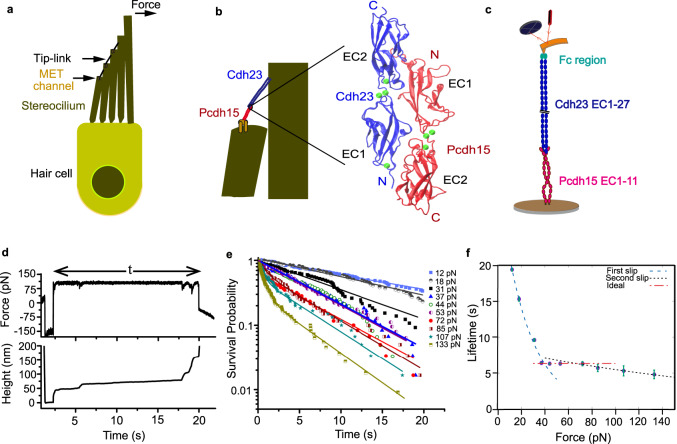
Ideal response of the tip-link *complex*. **a** Schematic of a hair-cell where each stereocilium in the hair bundle is connected to the next taller stereocilium by the tip-link. **b** The interacting domains (tip-link *interface*) in tip-links are highlighted. The zoomed region depicts the ribbon diagram of the tip-link *interface*. **c** Schematic of the AFM setup used for the force-clamp experiment of the tetrameric tip-link *complex*. Cdh23 EC1-27 (blue) with the Fc region (cyan) on its C-terminus is covalently attached to the cantilever, while the dimer of Pcdh15 EC1-11 (PICA) (magenta) is attached to the surface. **d** Representative time traces of force (upper) and height (lower), obtained from the force-clamp experiment. The bond-survival time is measured from the duration of clamp-time. **e** Bond survival probabilities for ten clamping forces (*n* = 701 events all-inclusive). Solid lines represent the exponential fit. Please see Supplementary Fig. 3 and Supplementary Table 1. **f** The tetrameric tip-link *complex* exhibits slip-ideal-slip bonds with force. Dotted lines are Bell model fits. Data are presented as fit values from (e) ± fitting error.

Tip-links exist as hetero-tetramer^5^. A cis-homodimer of Pcdh15 forms a fork-like structure with its two outermost extracellular (EC) domains (EC1, EC2) and facilitates trans-binding with two opposing Cdh23 proteins (Fig. 1a, b)^6,7^. The tail of the Pcdh15 dimer mechanically gates the MET channel^8,9^. Apart from EC1-2 domains at the tip-link interface, Cdh23 contains 25 additional EC domains, and Pcdh15 contains 9. These domains, topologically similar across tip-links, facilitate lateral interactions and may form cis-homodimers^10–12^. Apart from EC domains, there are linkers connecting neighboring domains. Linkers introduce kinks and bends in cadherin^13,14^. Kinks and bends in proteins are mechanical linkages that contribute to the elasticity of the tertiary structure^15,16^. In this text, we refer to the heterodimeric complex of a single Pcdh15 with a Cdh23 as an ‘*individual* tip-link’ (Supplementary Fig. 1a), while ‘tip-link *complex’* refers to the heterotetramer (Supplementary Fig. 1b), and ‘tip-link *interface*’ refers to the binding interface of an individual tip-link mediated by the EC1-2 domains (Supplementary Fig. 1a). Tip-links exploit their tertiary structure elasticity in response to mechanical stimuli and contribute to force-dissipation^17–20^. However, the conformational topography determined from electron microscopy and all-atom simulations projected tip-link complexes as being too rigid to act as gating-springs^6,21^. Therefore, additional viscoelastic molecules like ankyrin were recruited to elicit such behavior. Such additional elements intuitively lead to longer relaxation times^22^ and, thus fail to explain the observed short relaxation times of 10 µs or less at high auditory frequencies^3,23^.

For Homo sapiens, the perceived range of sound is 20 Hz-20 kHz in frequency and 5–120 dB in intensity, leading to a tension of 10–100 pN on tip-links at kHz loading rates^24,25^. The average lifetime of the tip-link complex under resting force (~10 pN) has been estimated to be ~8 s^26^. Moreover, the lifetimes of the tip-link drop exponentially with force when measured at low force-loading rates^26^. This indicates that in a nightclub or orchestra, hardly any tip-link complexes ought to survive, rendering most people deaf. Since this does not occur, it seems appropriate to expect a mechanism that safeguards the transduction at large forces. We aim to decipher the temporal force-response of the binding interface of the tip-link complex that enables it to survive forces of varying frequency and amplitude and capture the features that can explain uninterrupted hearing. The underlying hypothesis is that the force sensed at the tip-link *interface* is proportional to the force propagated to the MET channel.

We therefore clamp the tip-link *complexes* at constant tensile forces using an atomic force microscope (AFM) and monitor the response of the binding interface. The use of AFM is the key to achieving the higher force-loading rates equivalent to high-frequency, loud sound. In addition, we studied the force response of a mutant tip-link that is associated with congenital hearing loss. Moreover, existing genetic screening in model systems and other physiological studies suggest that Cdh23 may be the dominant elastic component of tip-links^20,27,28^. In this study, we also report on how alterations in protein elasticity as seen in different variants of Cdh23 affect the bond-lifetime dynamics of tip-links.

## Results

### Tip-link *complex* follows slip-ideal-slip bonds

Pcdh15 EC1-11 PICA exists predominantly as a cis-dimer^10,19^ (Supplementary Fig. 2). For the cis-dimer of Cdh23, we recombinantly tagged the C-terminal of the protein with the fragment crystallizable region (Fc-region) of an antibody. The Fc constructs self-ligate laterally and brings a pair of Cdh23 proteins in proximity (Methods, Supplementary Fig. 2). For survival lifetime measurements, we covalently attached the cis-dimers of Cdh23 EC1-27 at the cantilever and Pcdh15 EC1-11 PICA dimers on a coverslip (Fig. 1c), following a Sortase A-mediated transpeptidation protocol^29^ (Methods). We performed force-clamp experiments for a range of forces and estimated the lifetime of the tip-link *complex* from each successful clamping, as shown in Fig. 1d. From the distribution of lifetimes, we calculated the survival probability (SP) (see Methods) of the *complex* for each clamping force (Fig. 1e & Supplementary Fig. 3). Subsequently, we obtained the lifetimes of the tip-link *complexes* at varying clamping forces (see Methods) and plotted the force-lifetime relation in Fig. 1f. It is important to note that for every force-clamp experiment, we performed control experiments to quantify the non-specific contribution to the measurements (see Methods).

Our results indicate that the tip-link *complex* shows three distinct types of responses as a function of the clamping force (Fig. 1f, see Methods). In the low-force regime, the complex displays conventional slip bond behavior, where the lifetime decreases monotonically with increasing force. For an intermediate range of forces (~36 pN to ~70 pN), the tip-link *complex* displays an ideal-bond character (Fig. 1f) of being indifferent to a range of tensile forces. Forces beyond 80 pN, which correspond to extreme auditory inputs, cause the tip-link *complex* to progressively dissociate, exhibiting a second regime of slip bond response.

Extrapolating the lifetime in the low-force regime to zero force yields an intrinsic lifetime (τ_0_) of the tip-link *complex* to ~31.8 s in the ballpark of the reported values (22 s–63 s), estimated from the optical tweezer measurements as well as BLI experiments^26^. We also observed that the survival probability of tip-link *complexes* at high forces (>70 pN) follows bi-exponential decays with time (Fig. 1e & Supplementary Fig. 3) instead of mono-exponents as in low-force regions (Supplementary Table 1). The emergent component is short-lived (~1 s) (Supplementary Fig. 4) and may be associated with dissociations of *individual* tip-links without rebinding, which otherwise would have enhanced the lifetime. This is also reflected in the corresponding amplitude, which increases with increasing clamp-forces (Supplementary Fig. 4). Lesser rebinding probability at higher tensile forces has also been reported previously for tip-link *complexes*^26^.

We noticed brief and sudden spikes in force-clamp traces where the force momentarily drops with a spike duration of ~0.16 ± 0.01 s. These spikes mark the corresponding extensions of tip-link *complexes* under tension, which can be quantified from the sudden jumps in the tip-surface distances or heights (Fig. 1d). Statistically, we observe that an extension of 5.3 ± 0.3 nm is the most frequent, followed by an extension of 11.5 ± 0.2 nm, across the entire force range with significantly larger extension events occurring at intermediate forces (Supplementary Fig. 5). While similar conformational alterations were reported previously for Pcdh15 alone^17^, our observations indicate comparable elongations in the tip-link *complexes* under tension. Further, the first extension is nearly instantaneous in nature across the entire force-clamp range (Supplementary Fig. 6). While elongations in tip-link complexes have been associated with their gating-spring properties in the literature^17,30^, we have not been able to determine alterations in the effective stiffness from our data.

### Tip-link *interface* follows slip-catch-slip bonds

**Fig. 2:**
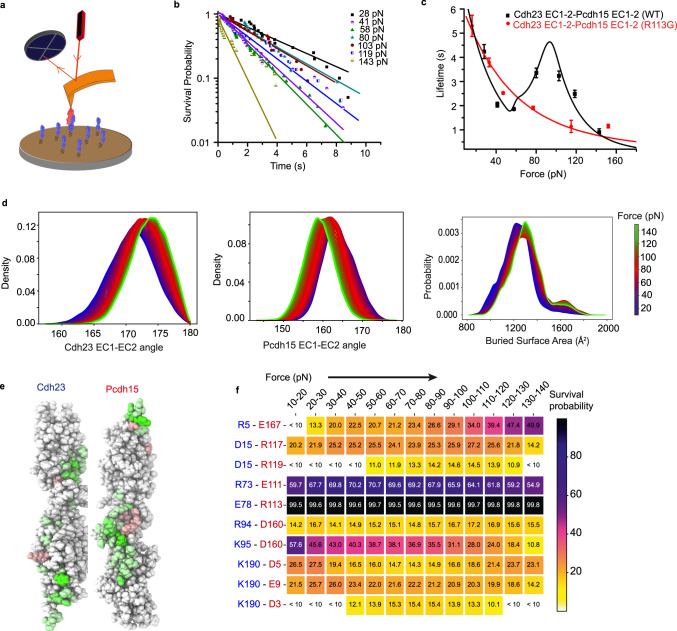
Slip-catch-slip response of the tip-link *interface*. **a** Schematic of the force-clamp experiment where Pcdh15 EC 1-2 (red) is attached to the cantilever and Cdh23 EC1-2 (blue) is onto the surface. **b** The bond-survival probabilities for the tip-link *interface* are shown here for the seven clamping forces of 28, 41, 58, 80, 103, 119 and 143 pN containing *n* = 20, 29, 57, 24, 23, 23, and 26 datapoints, respectively. Solid lines represent the exponential-decay fit. **c** The dependence of bond-lifetime on clamping forces is shown here for wild-type (WT, black squares) and R113G-mutant (red squares). The WT shows tri-phasic slip-catch-slip behavior. Data are presented as survival probability fit values from (**b**) ± fitting error. Black and red solid lines are the kinetic model fits (see Method). **d** Distributions of inter-domain angles between EC1 and EC2 of Cdh23 (left) and Pcdh15 (center) at the 61 interpolated points between the force range of 10 and 150 pN from FISST simulations. Variations in inter-domain angle are accompanied by a change in buried surface area (right). (*n* = 3 independent simulations, see Supplementary Fig. 7). **e** The per-residue differential buried surface area is calculated by subtracting the buried surface area at 10–20 pN force range from the 70–80 pN force range. The residues with a positive differential buried surface area are colored green, with darker shades representing higher values. The residues colored as red show a negative differential buried surface area, with darker shades representing larger negative values. **f** The percentage of frames showing the existence of specified salt-bridges at different force ranges. Notably, the salt-bridge R113-E78 persists at all forces. See also Supplementary Figs. 8–11.

The two outermost domains of cadherins (EC1-2) overlap with each other over a surface area of ~1000 Å^2^ and form a tip-link *interface*^7^ (Supplementary Fig. 1). To decipher the molecular origin of the slip-ideal-slip response of tip-link *complexes*, we first set out to study the force-lifetime dynamics of the tip-link *interface* (Fig. 2a, see Methods). When we measured the bond-survival dynamics of the *interface* (Fig. 2b), we obtained a tri-phasic slip-catch-slip bond behavior without any ideal bond regime (Fig. 2c). Initially, the *interface* lifetime drops monotonically with increasing force as a slip bond response. Above a critical force (F_C1_) of 58 pN, the bond-lifetime begins to increase with force and reaches a maximum at a critical force (F_C2_) of 80 pN, referred to as a catch-bond. As we show in the next section, the catch bond leads to a stronger interface under mechanical perturbation. Beyond 80 pN, the lifetime again decreases monotonically with force reflecting slip behavior.

### Molecular details shed light on the slip-catch-slip transitions

The striking difference in the force-response of the tip-link *interface* in comparison to the *heterotetramer* complex raises several fundamental questions. What is the molecular mechanism of the slip-to-catch transition of the *interface*? How do the slip-catch-slip bond dynamics of the *interface* manifest in the force response of an *individual* tip*-*link? What is the role played by molecular elasticity? How does the slip-ideal-slip bond of the *complex* emerge from the dynamics of the *individual* tip-links?

To systematically study these questions, we performed Steered Molecular Dynamics simulations (SMD) of the *interface* in a single tip-link using the FISST (infinite switch simulated tempering in force) method^31^. We monitored the effect of tensile forces on the *interface* by tracking the residue-wise interactions from changes in the interdomain angles, bound surface area, and binding energy (see Methods). Like in the force-clamp experiments, we applied constant forces of 10 − 150 pN using the **X** component of the end-to-end distance as the collective variable (with the long axis of the complex aligned to the X axis). During simulation runs, the tip-link interface remained stable under these forces for microseconds, indicating no complex dissociation or domain-unfolding. Rather, the external force tilts the energy landscape, which is sampled by FISST simulations. Observing such changes without FISST would have required much longer cumulative simulation time. We observed an emergent population of conformations with a distinctly larger buried surface area at intermediate forces (Fig. 2d), and accordingly, increased binding energy of the two proteins, calculated using the MM-GBSA approach, as a function of force (Supplementary Fig. 7). Calculating the differential per residue buried surface area for the force range of 70–80 pN (compared to 10–20 pN), we noted that residues in both the EC1 and EC2 domains, as well as the N-termini of both proteins, are buried deeper upon the application of force (Fig. 2e). To map the inter-protein interactions that are *newly* formed or strengthened under tension, we calculated the survival probability of the salt-bridges (Fig. 2f), H-bonds (Supplementary Fig. 8), and hydrophobic interactions (Supplementary Fig. 9) between Cdh23 and Pcdh15 within the simulation time. An increase in buried surface area implies the strengthening of the tip-link *interface* in response to applied forces, a molecular picture of catch bonds. Unlike in the experiments, we did not observe any SMD feature of slip-bonds for tip-link *interfaces* in the low-force regime. Perhaps an initial encounter complex is formed in the force-clamp experiments, which settles into a conformation similar to the crystal structure at very low forces. If this relaxation dynamics is the source for slip-bond behavior, then it will be absent in simulations because we start from the crystal structure.

### The salt-bridge that serves as molecular fulcrum

However, we identified a striking salt-bridge interaction between Pcdh15(R113) and Cdh23(E78) that persisted throughout the SMD (Fig. 2f, black shade). Analysis of force-storage and force-transmittance probability (see Methods and Supplementary Text) of bonds further showed constant mechanical tension between the R113-E78 salt-bridge across the applied force range (Supplementary Fig. 10a, b). This bond, which is unconventionally impassive over a large range of tensile forces, may be described as a pivot or molecular fulcrum. We determined the force distributions among residue-pairs connected to the molecular fulcrum through space (inter-protein) (Supplementary Fig. 10c, d) and covalent linkages (intra-protein) (Supplementary Fig. 11). This allowed us to identify multiple residue-pairs that reconfigure their bond-mechanics to redirect the external tension away from the fulcrum (see Methods and Supplementary Text). Based on our simulations, we propose that this pivot is key to strengthening the interface under the applied tension, as implied by the previous discussion.

### Mutation in Pcdh15 (R113G) abolishes the slip-to-catch transition

The importance of R113 in Pcdh15 is independently known in physiology. A mutation of R113 to glycine (R113G) is prevalent in patients suffering from inherited deafness^32^. It is, therefore, interesting to study the bond-lifetime dynamics of *individual tip-links* containing the R113G mutant. Accordingly, we recombinantly synthesized Pcdh15 EC1-2 (R113G) and repeated the force-clamp measurements. The lifetime-force curve of the mutant is dramatically different, featuring a slip bond exclusively (Fig. 2c). Thus, we may infer that the loss of the pivot adversely affects the formation of catch bonds in individual tip-links, and so the hearing. These outcomes obtained with the mutant point out that the slip-catch-slip feature of the WT tip-link could be essential for normal hearing.

Though the nature of the force-lifetime dynamics of the mutant *interface* differs from the WT *interface*, the lifetime in the low-force regime is comparable for the two variants (Fig. 2c). Taken together with the observation that the R113G variant loses the catch response, we infer that the pivot plays a significant role in the high-force regime and therefore is instrumental in the catch bond region. To quantify the role of the pivot, we constructed a kinetic model to describe the bond-lifetime dynamics of WT and mutant *interfaces*.

### A kinetic model to explain the slip-catch-slip transitions

A dynamic switch in the stiffness of biological bonds under tension was first hypothesized as responsible for slip, catch, and ideal bonds^33^. Subsequently, several phenomenological theories, including a two-pathway model^34^, allosteric bond model^35^, deformation model^36^, sliding-rebinding model^37^ were constructed to model the catch bonds. From the SMD studies presented in the preceding section, we have learned that force-induced tilting to a stronger binding interface through a pivot appears to be responsible for the catch bond. Conceptually, the force-induced switch closely resembles the ‘sliding-binding model’^37^. We, therefore, modified the sliding-rebinding model to incorporate the slip-catch transition and fit our data (see Methods). For the mutant tip-link interface that displays only slip-bond dynamics, the pivotal action is lost, and thus, the stronger binding interface is not formed. The fits of the model to our experimental data for the WT and mutant tip-link interfaces are shown in Fig. 2c, and the parameter values are presented in Table 1. We obtain comparable values for the intrinsic off-rate, ${k}_{-1}^{0}$ and distance to transition, *x*_*β*_ for both WT and mutant tip-link interfaces, indicating that the initial bound-states for WT and mutant interfaces are nearly identical under no tension. External forces tilt the bound-state towards dissociation featuring slip bonds.

**Table 1:** Kinetic parameters obtained from the modified sliding-rebinding model fit of lifetime-force data for the tip-link *interface* and its mutant variant

*Interface*	*k*_-1_ (s^−1^)	*x*_*β*_(Å)	*k*_+1_ (s^−1^)	*k*_+2_ (s^−1^)	*F*_*C*2_ (pN)	*n*
WT	0.5 ± 0.4	1.0 ± 0.5	2.8 ± 3.6	30.2 ± 38.8	100.0 ± 9.4	2.3 ± 1.6
Mutant	0.3 ± 0.1	0.6 ± 0.1	0.6 ± 0.4			

### Molecular stiffness of cadherins in the force-lifetime dynamics

**Fig. 3:**
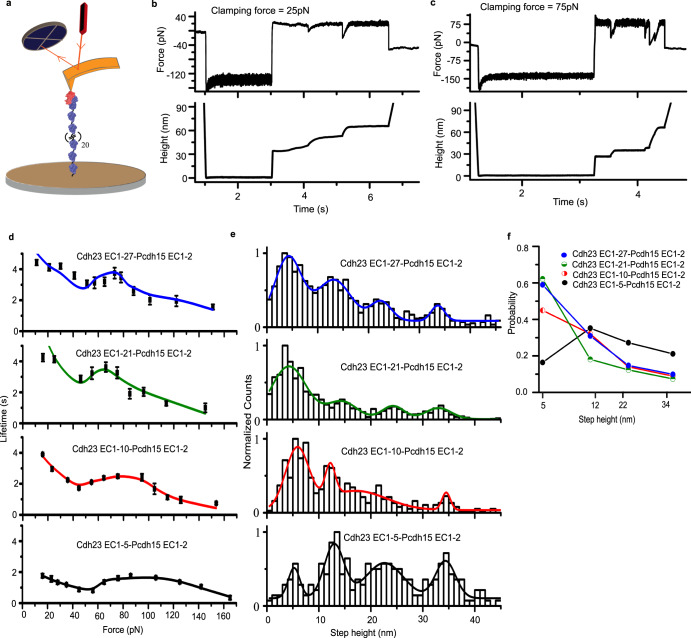
Force-lifetime trends for the tip-link variants. **a** Schematic representation of an AFM-based force-clamp experiment for *individual* tip-links. **b**, **c** Representative time traces of force (upper) and height (lower). **d** The lifetime-force behavior of the tip-link variants. All the variants depict a slip-catch-slip transition with increasing force. Data are obtained from the single exponential decay fitting of the survival probabilities (*n* = 577 events for Cdh23 EC1-5-Pcdh15 EC1-2, 628 events for Cdh23 EC1-10-Pcdh15 EC1-2, 376 for Cdh23 EC1-21-Pcdh15 EC1-2, and 1016 events for Cdh23 EC1-27-Pcdh15 EC1-2, inclusive of all forces, see Supplementary Figs. 12–15) ± fitting error. Solid lines are the modified sliding-binding kinetic model fit. **e** Unfolding step-height distributions, calculated from all the force curves for all Cdh23 variants, Cdh23 EC1-5 (black) (*n* = 226 datapoints), Cdh23 EC1-10 (red) (*n* = 436 datapoints), Cdh23 EC1-21 (green) (*n* = 1108 datapoints), and Cdh23 EC1–27 (blue) (*n* = 1788 datapoints). Solid lines are the Gaussian fits to the distribution, providing the most probable step-height from the peak maxima. **f** Relative probabilities of each type of step-height change for clamp measurements with varying lengths of the Cdh23 construct. See also Supplementary Figs. 12–17.

Dynamic alterations in the conformations of the long tip-link cadherins under tension have already been reported^17–20^. How such alterations contribute to the bond-lifetime dynamics of the *individual* tip-link is not yet clear. To achieve this, we systematically altered the molecular stiffness of *individual* tip-links by varying the number of non-interacting domains and studying the survival times. Cdh23 possesses a larger number of conformations and the least axial stiffness among the tip-link proteins. Therefore, we recombinantly synthesized four variants of *Cdh23*: Cdh23 EC1-5 (5 domains), Cdh23 EC1-10 (10 domains), Cdh23 EC1-21 (21 domains), and full-length Cdh23 EC1-27 (27 domains) (Methods). The stiffness of Cdh23 monotonically drops with an increasing number of domains and linkers^38^. For the force-clamp experiments, we used the two outermost domains of Pcdh15 (EC1-2) as the pulling-handle by covalently attaching to the cantilever using sortagging method. Cdh23 variants were attached independently and covalently to the coverslip (Fig. 3a, Methods). The slip-catch-slip bond features persist in all variants (Fig. 3d), though the overall bond lifetimes of the variants (Supplementary Fig. 12–15) increased with increasing domain numbers (Fig. 3d). Thus, we conclude that the slip-catch-slip behavior is a trademark of the tip-link *interface*, and molecular stiffness alone does not alter this feature.

We also observed four characteristic extensions of the Cdh23 protein (~5 nm, ~12 nm, ~22 nm, and ~34 nm) in all four variants, prior to dissociation (Fig. 3b, c, e and Table 2). Among these, extensions of ~5 nm and ~12 nm are most frequent (Fig. 3f). Interestingly, the ~5 nm extension, referring to inter-domain linker elongation, has already been reported as elastic stretching^17^. Our force-distribution map of the SMD simulations also captured the linkers as pre-compressed between the EC1-2 domains of both proteins (Supplementary Fig. 10). Finally, it is tempting to infer a step increment in the percentage of unfolding-associated unbinding events as we proceed from EC1-5 and EC1-10 to EC1-21 and EC1-27 **(**Supplementary Fig. 17). Structural elucidations of Cdh23 fragments reveal a single non-canonical linker in EC1-5 and EC1-10, and the rest are beyond EC10 (this was how we engineered these proteins)^11^. This could be the underlying reason for the step-increment since domains connected with non-canonical linkers attain lesser stability and preferentially unfold under tension. A similar trend can also be seen in the kinetic parameters in the Table 3. More importantly, the extensions, ~5 nm and ~12 nm, in *individual* tip-links are comparable to the elongations we obtained for the tip-link *complexes*, indicating that the elastomeric components in tip-link proteins are preserved in the tetrameric conformation of the complex.

**Table 2:** Most probable step heights from peak maxima of the unfolding step height distributions for *individual* tip-link variants

Variants	Step height 1 (nm)	Step height 2 (nm)	Step height 3 (nm)	Step height 4 (nm)
Cdh23 EC1–5	5.2 ± 0.3	12.9 ± 0.3	22.6 ± 0.5	34.3 ± 0.3
Cdh23 EC1–10	5.8 ± 0.3	12.2 ± 0.2	17.1 ± 2.9	34.4 ± 0.9
Cdh23 EC1–21	3.7 ± 0.2	10.0 ± 0.6	24.6 ± 0.7	32.8 ± 0.8
Cdh23 EC1–27	4.2 ± 0.2	13.2 ± 0.4	22.4 ± 0.4	33.6 ± 0.4

**Table 3:** Kinetic parameters obtained from the modified sliding-rebinding model fit of lifetime-force data for *individual* tip-link variants

*Variants*	*k*_-1_ (s^−1^)	${x}_{\beta }$(Å)	${k}_{+1}$ (s^−1^)	${k}_{+2}$ (s^−1^)	${F}_{C2}$ (pN)	*n*
Cdh23 EC1–5	0.61 ± 0.15	1.2 ± 0.1	0.9 ± 1.1	29.4 ± 16.0	108.4 ± 4.7	1.2 ± 0.3
Cdh23 EC1–10	0.39 ± 0.11	1.0 ± 0.1	0.9 ± 0.5	7.8 ± 3.8	99.0 ± 6.2	1.9 ± 0.6
Cdh23 EC1–21	0.35 ± 0.18	0.7 ± 1e-5	1.2 ± 1.9	0.8 ± 1.4	72.0 ± 20.7	3.8 ± 6.8
Cdh23 EC1–27	0.29 ± 0.03	0.6 ± 1e-4	0.5 ± 0.1	0.5 ± 0.2	86.8 ± 10.1	3.0 ± 1.9

Notably, such extensions of classical cadherins under tension are rarely reported in the literature. The topologies of the EC domains in the classical cadherins and the non-classical tip-link cadherins are comparable. Tip-link cadherins, however, possess numerous non-canonical interdomain linkers that are absent in classical cadherins^11^. Such linkers adversely affect domain stability and, intuitively, may contribute to force-induced extensions.

We take into account the unfolding kinetics in the tip-link survival by including the extension probabilities (Fig. 3e) obtained from experiments in our kinetic model **(**see Methods). We fitted the lifetime-force data for all the extensions for full-length Cdh23 EC1-27 to a single exponential decay and obtained the corresponding zero-force unfolding rates (${k}_{u}^{0}$) and distances to transition-states (${x}_{\beta }^{u}$) (Supplementary Fig. 18, Table 4). For simplicity, we considered the two most-probable extensions of ~5 nm and ~12 nm. Using these extension probabilities, we fit the bond dissociation dynamics of the variants (Fig. 3d, see Methods). The zero-force lifetime increases from ~1.6 s for the Cdh23 EC1-5 to ~3.4 s for the Cdh23 EC1-27 variant. The critical force (F_C2_) at which the catch switches to slip bonds shifts lower, from 108.4 ± 4.7 pN for Cdh23EC1-5 to 86.8 ± 10.1 pN for Cdh23 EC1-27. Further, we noted a decreasing trend in the distance to transition state (*x*_*β*_) and rebinding-rate (*k*_+2_) with increasing domains (Table 3). However, the parameter referring to the force-enhanced binding, *n*, increases with the domain number, with the smallest value of 1.2 ± 0.3 for 5 EC domains to the highest value of 3.0 ± 1.9 for 27 EC domains. This increase in *n* enhances the force-resistance of tip-links against dissociation (higher bond-lifetime). The lowering trend in *x*_*β*_ too signifies higher force-tolerance with an increase in domains. The reorganization rate, (*k*_+1_), of the binding interface, however, remains unaffected with increasing domains. In short, all the fitting parameters indicated that increasing domain numbers did uplift the bond-survival significantly under moderate tension (Fig. 3d). However, the journey from slip-catch-slip bonds of *individual* tip-links to slip-ideal-slip bonds for tip-link *complexes* is still not clear.

**Table 4:** Kinetic parameters obtained from Bell’s-model fitting of force-dependent lifetime data of *individual* step height in Cdh23 EC1-27-Pcdh15 EC1-2 complex

Step height gain	4.2 ± 0.2 nm	13.2 ± 0.4 nm	22.4 ± 0.4 nm	33.6 ± 0.4 nm
${x}_{\beta }$(Å)	2.3 ± 0.4	1.0 ± 0.2	1.8 ± 0.6	0.7 ± 0.1
${k}_{-1}^{u}$ (s^−1^)	0.17 ± 0.06	0.45 ± 0.06	0.45 ± 0.15	0.36 ± 0.03

### Tetrameric assembly of tip-links is essential for slip-ideal-slip bonds

Since the slip-ideal-slip feature is exclusive to the tip-link *complex*, it is logical to hypothesize that the switch from slip-catch to slip-ideal bonds may be steered through dimerization of independent tip-links. While the SMD simulations provided an extensive understanding of the nature of the catch mechanism in *individual* tip-link *interfaces*, such simulations are prohibitively expensive for the tetramer. However, coarse-grained simulations that incorporate catch-bond behavior using phenomenological theories have provided valuable insight into the behavior of a load-sharing cluster of such bonds^39–43^.

**Fig. 4:**
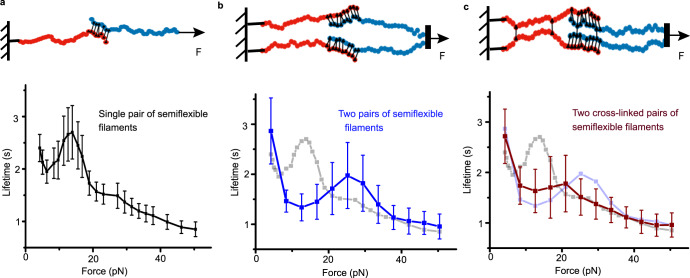
The coarse-grained Langevin dynamics simulations of coupled polymeric systems. **a** Schematic representation (top) of two semiflexible filaments coupled with multiple elastic slip-catch bonds resembling the *individual* tip-link, and clamped at constant forces to perform the coarse-grained Langevin dynamics simulations. Bottom curve shows the resulting force-dependent lifetime that depicts the slip-catch-slip behavior. **b** Schematically representing the two pairs of semiflexible polymers coupled and pulled in combination at constant forces. The slip-catch-slip feature remains unaltered (blue curve, bottom) for this polymer system; however, lifetime in the catch-regime is dropped compared to single pair of filaments (grey curve in background). **c** A modified polymer model from (**b**) where the chains from the same sides are connected. This model mimics the heterotetrameric geometry of the tip-links. The characteristic force-lifetime curve exhibits a slip-ideal-slip feature (dark red, bottom). Grey and light blue curves in the background are from models (a) and (b). Error bars represent standard errors, *n* = 100 simulations per data-point. Data for (**a**–**c**) are presented as mean values ± SEM. See also Supplementary Fig. 19.

Therefore, we performed simulations of *coupled* polymeric systems in the presence of noise (see Methods). We first considered an arrangement of two semiflexible filaments partially attached to one another via elastic bonds with catch-slip dissociation characteristics (see Methods, Fig. 4a). The bonds dissociate in the presence of a load when one of the filaments is pulled longitudinally. The average lifetime of the attachment, defined as the time beyond which all bonds between the two filaments break, leading to two separated filaments, is plotted as a function of the external force (Fig. 4a, Supplementary Movie 1). Unsurprisingly, the dimeric tip-link arrangement consisting of multiple bonds showed slip-catch-slip behavior in the average lifetime versus force. The coarse-grained parameters of the model were chosen to ensure that the ranges of lifetime and force were consistent with those observed in the experiments.

We next considered the tetrameric arrangement as shown in Fig. 4b. Here, two filaments from each of the dimeric arrangements are pulled at one end simultaneously. We first assumed that there are no elastic bonds cross-linking the two dimeric systems, except for the end monomers, which are both held at the same position. The force-response of the bonded interface still follows a slip-catch-slip characteristic, although this feature is significantly reduced in the tetrameric arrangement, with lifetime-maxima shifting to higher force values. This shift in the lifetime maxima can be explained based on the sharing of the external load force between the two dimeric systems. The force acting on each dimeric arrangement is halved, which would explain why the maxima in lifetimes now appear at roughly double the force in this tetrameric arrangement. Further, the increased entropy of the tetrameric arrangement due to the excluded volume interactions results in a faster detachment of the elastic bonds. This leads to a significantly reduced lifetime when compared to the dimeric system for almost all forces.

We then cross-linked the polymers that are anchored in a manner similar to the cis-dimers of Pcdh15 (Left side chain in Fig. 4c, Supplementary Movie 2). Additionally, Cdh23 interacts laterally through their EC1 domains in the tetrameric complex^19^. Accordingly, we cross-linked the polymer pairs that are being pulled at their cross-linking *interface* (Right side chain in Fig. 4c). The cross-linking flattens the catch-region, leading to slip-ideal-slip behavior. This phenomenon persists over a range of values of model parameters (see Methods). Thus, the slip-ideal-slip behavior appears in our coarse-grained simulations as a cooperative effect brought about by the lateral cross-linking of *individual* tip-links.

## Discussion

With the overarching objective of deciphering the force-transduction mechanism of the tip-links, we devised careful and detailed studies that probed the propagation of force through the tip-link complex. The most important result of our studies is that the tip-link *complexes* exhibit a slip-ideal-slip type of response to applied forces.

Surprisingly, the slip-ideal-slip bond is not intrinsic to the *individual* binding interfaces of a tip-link complex. Rather, these obey slip-catch-slip dynamics, with the catch region appearing as a region of increasing lifetime with applied force (Fig. 2c). Based on evidence from SMD simulations, we hypothesize that the catch regime appears due to a force-induced increase in the buried contact surface area between tip-link proteins, mediated by a pivot salt-bridge (Pcdh15(R113) - Cdh23(E78)) **(**Fig. 2d, f, & Supplementary Figs. 7–9). A careful analysis of the force fields identified several force-disseminating routes diverging from the pivot and making the salt-bridge force-resistant towards dissociation (Supplementary Fig. 10). We show that the mutation, R113G, in Pcdh15 that is associated with congenital deafness removes the catch region dynamics of the *individual* tip-link. A kinetic model allowed us to obtain parameters associated with the slip-catch-slip dynamics of *individual* tip-links. We then examined the effect of molecular elasticity by varying the length of the tip-link protein, Cdh23, without affecting the interface and studied the force response. We observe no change in the overall nature of the bond-survival dynamics with increasing length, except for an increase in the overall lifetimes (Fig. 2c). Finally, using molecular Langevin dynamics, we show that the slip-ideal-slip dynamical response can emerge from the dimerization and cross-linking of the *individual* tip-links exhibiting catch bond dynamics (Supplementary Fig. 19) as in the actual tip-link complex, at least for a range of model parameters.

Catch bond, a mechanism to withstand force-induced dissociations, is not rare in biology and is even known for classical cadherins^44^. Though we report the slip-catch-slip dynamics for *individual* tip-link interfaces, a recent force-ramp study using optical tweezers (OT) reported slip dynamics for the same^26^. We noted that the ramp-rate in OT was 0.39-271.5 pN/s, significantly slower than the average rate used in our experiments (88,000-119,200 pN/s). This difference in the nature of the bond dynamics may arise from the loading-rate mismatch. While a very slow loading of force may be unable to capture the tilting or sliding event, a very fast loading rate can bypass the sliding to a stronger binding interface^45,46^. In both cases, the bond-lifetime dynamics appear to slip.

Tonotopy of mammalian cochlea is associated with the intrinsic stiffness of tip-links^17,47^. The stiffness increases with increasing frequency, and so does the resting tension on tip-links. It is estimated that tip-links have a stiffness of 1.3 pN/nm and experience a resting tension of 5 pN at 1 kHz. At a higher frequency of 4 kHz, the stiffness and the resting tension reach 3.7 pN/nm and 34 pN, respectively. Our study with the varying lengths of tip-links reveals that the intrinsic bond-dynamics of the *individual* tip-link interface do not alter with stiffness. The survival of the interface, however, is adversely affected by increasing tip-link stiffness (lower lifetime with shorter domain lengths), indicating a diminishing lifetime of *individual* tip-links with increasing frequency. However, determination of the true effect of *oscillatory* force fields on the survival dynamics of the tip-link complex requires further investigation.

In this work, we have shown the unique transition of a catch-binding interface to an ideal bond. In the literature, ideal bonds have been generally referred to as experimental artefacts where the direction of force application and bond dissociation are orthogonal to each other^34,44^. However, we have argued that the ideal bond emerges from the lateral dimerization of the catch interfaces of *individual* tip-links only when the interprotein couplings between two Pcdh15 and two Cdh23 are established (Supplementary Fig. 19). For Pcdh15, these interprotein lateral couplings are at the PICA domain and EC3 domain, away from the interacting interface. For Cdh23, such lateral coupling is formed between EC1 domains only when in proximity in the heterotypic tip-link tetramer geometry.

Due to the lateral links, the applied forces can be expected to be dispersed in the constituent proteins (leading to structural rearrangements) and only partially conveyed to the MET terminal interface. Hence, the presence of an ideal bond could be regarded functionally as a force-block filter in analogy to frequency filters used for electromagnetic waves. This is supported by the following observations: (a) The ideal response occurs in the range of forces beyond normal auditory loudness (assuming normal is below 40pN^26^) where we expect a protective mechanism to be operating. (b) In the initial slip region, the rapid variation in lifetime with force is indicative of heightened sensitivity to changes in auditory pressure. This might be a desideratum for normal auditory transduction. (c) Beyond the ideal regime, nearly total dissociation of the tip-links is expected to occur. This may be correlated with significant hearing loss from loud sounds. (d) The increase in the number of non-canonical linkers is seen to increase the lifetimes (Fig. 3d). This could be interpreted as a partial dissipation of the applied force in the unfolding events at these domains (Supplementary Fig. 16). In fact, Supplementary Fig. 5 shows that a significantly larger number of unfolding occur in the ideal regime.

We also note that in our Langevin dynamics simulations, ideal response emerges by cross-linking the dimers which can be expected to increase the rigidity of the complex. Increased rigidity can lead to nearly complete force-transmission - although this does not necessarily imply increased sensitivity. In this case, the MET channels are exposed to the entire input stimulus.

Establishing a firm connection between the survival lifetimes and the transmitted forces will resolve this dichotomy. This may be possible through further simulations and suitably modified experiments which can characterize the distribution of the applied forces through the complex.

## Methods

### Cloning, expression, and purification of Cdh23 EC1-2 and Pcdh15 EC1-2 using bacterial system

Mouse Cdh23 EC1-2 and Pcdh15 EC1-2 were recombinantly modified with 6xHis-tag at N-terminal for affinity-purification, and sort-tag (LPETG) at C-terminus for surface attachment and cloned into Nde1 and Xho1 sites of pET21a vector. Mutation R113G in Pcdh15 EC1-2 was engineered using overlapping extension PCR. All the constructs were verified by DNA sequencing. Proteins were expressed in *E.coli* BL21 Codon Plus (DE3)-RIPL cells (Stratagene, San Diego, CA, USA) cultured in LB media. Cells were induced with 200 µM IPTG at OD 0.6 at 16 °C for 16 h. Cells were lysed by sonication in the 8M urea buffer and protein was purified from inclusion bodies using Ni-NTA based affinity purification. Eluted proteins were refolded by sequential dialysis in decreasing concentration of urea and further purified with size exclusion chromatography. The purity of the proteins was monitored from SDS-PAGE gel electrophoresis.

### Cloning, expression, and purification of Cdh23 variants in mammalian system

For the Cdh23 EC1-27 construct, we ligated the two fragments of Cdh23 having overlapping regions using Gibson assembly^48^. From the Cdh23 EC1-27, we generated all the truncated variants; EC1-5, EC1-10, and EC1-21 between restriction sites NheI and XhoI in vector pcDNA3.1 (+) using the DNA recombination method. All the constructs have sort-tag (LPETGSS), GFP and 6xHis-tag at the C-terminus. Sort-tag was inserted for covalent attachment to the surface, GFP tag helped in tracking the protein expression and His-tag was necessary for affinity-based protein purification. All the constructs have signal peptides at the N-terminus to guide the expressed protein in the media.

For the heterotetrameric tip-link construct, we recombinantly modified the C-terminus of Cdh23 EC1-27 with a fragment crystallizable region (Fc-region) of a human antibody. The Fc constructs can self-ligate via disulfide linkages and bring a pair of Cdh23 proteins in proximity. For Pcdh15 EC1-11 PICA, we PCR amplified the entire Pcdh15 plasmid up to the PICA domain using specific primers and cloned in pcDNA 3.1 (+) vector between the restriction sites KpnI and XhoI.

For protein expression, we used Expi-CHO suspension cells (Thermo Fisher Scientific). Cells were transfected at the cell density of 10^8^ with the 1 µg/mL plasmid as the prescribed protocol and kept at 37 °C, 6% CO_2_, and 125 RPM in the incubator. After 20 h of transfection, feed and enhancer were added to increase the yield of protein expression. Media was collected after 7 days from the transfection and centrifuged at 520 × *g* for 10 min. After centrifugation, we dialyzed the supernatant media in the buffer (25 mM HEPES, 150 mM NaCl, 50 mM KCl, 5 mM CaCl_2_) at 4 °C. Dialyzed media carrying the protein of interest was purified using Ni-NTA based affinity purification.

### Surface modification for single-molecule force spectroscopy using AFM

Glass coverslips were activated using air plasma and subsequently washed with piranha solution for 2 h, followed by a thorough wash with deionized water. Coverslips were then etched using 1 M KOH for 15 min and washed with deionized water by sonicating for 10 min three times. Subsequently, the surfaces were silanized using v/v 2% APTES (3-Aminopropyltriethoxy silane) in 95% acetone and cured at 110 °C for 1 h. The amine exposed surfaces were reacted with 10% Maleimide-PEG-Succinimidyl ester (Mal-PEG5000-NHS) in methoxy-PEG-Succinimidyl ester (mPEG5000-NHS) (LaysonBio) in a basic buffer (100 mM NaHCO_3_, 600 mM K_2_SO_4_, pH 8.5) for 4 h for the experiment with just two interacting domains. For experiments with the varying lengths of Cdh23, we have used Mal-PEG2-NHS (Sigma-Aldrich) instead of (Mal-PEG5000-NHS). The PEGylated surfaces were subsequently incubated with 100 µM polyglycine peptide, GGGGC, at room temperature (RT) for 7 h for cysteine – maleimide reaction. Polyglycine on coverslip acts as a nucleophile for sortase-based covalent attachment. Coverslips were then washed with water and stored in a vacuum desiccator prior to protein attachment.

We used less stiff Si_3_N_4_ cantilevers (NITRA-TALL from AppNano Inc., USA) for all the force-spectroscopy studies. Cantilevers were functionalized in the similar way with GGGGC to attach Pcdh15 EC1-2 covalently using sortagging.

### AFM based force–clamp spectroscopy

C-terminus of Cdh23 was covalently immobilized on the freshly prepared glass-coverslips, coated with polyglycine. Pcdh15 was attached on cantilevers. Attachment of proteins on both cantilevers and coverslips were done using sortagging protocol, in the presence of enzyme sortase A^29^. Force-clamp spectroscopy was performed using Atomic Force Microscope (AFM) (Nano wizard 3, JPK Instruments, Germany). The spring constant of the cantilever was determined using equipartition-based thermal fluctuation method^49^, prior to each experiment. In force-clamp measurements, the cantilever was approached towards the surface to interact with its partner. We let the proteins interact for 1–2 s, thereafter, retracted the cantilever by 20–70 nm depending on the proteins attached to cantilever, and clamped at a certain force for 10 s until the bond dissociation occurred, and then finally retracted the cantilever to break all the remaining bonds formed. For experiments with Cdh23 EC1-27 Fc dimer, we retracted the cantilever 70 nm up prior to clamping and set a clamping time of 20 s. The retraction of 70 nm was done using the length-clamp mode of the closed-loop z-piezo. This step helps us to reduce the non-specific surface interactions. All the experiments have been performed in the buffer with 25 mM HEPES, 100 mM NaCl, 50 mM KCl, and 50 µM CaCl_2_. We repeated the experiment at different points on the surface and recorded around 2000 force-curves at each clamping force to get statistically significant data.

For the control experiment, force-clamp data was recorded in a no-calcium buffer: 25 mM HEPES, 100 mM NaCl, 50 mM KCl along with 1 mM EGTA. We noticed a sharp drop in the event rate to ~0.5–1% from 6–8%, indicating a negligible contribution from the non-specific interactions in our measurements. Further, we performed force-clamp measurements by attaching the protein in either of the two surfaces, like cadherins on the coverslip and no-protein to cantilever (coated with polyglycine alone). We obtained less than 0.4% of events in such measurements, reconfirming negligible contribution of non-specific data in our measurements. The specificity of the experiments was also justified by the measurements with the mutant Pcdh15 R113G tip-link which resulted in the only-slip bond behavior. Further, interaction between multiple proteins has been avoided by controlling the percentage of functional groups during the PEGylation of surface and cantilever. We have maintained the percentage as ~5–10% of surface and cantilever to perform all the experiments.

### Data analysis

After data acquisition, we measured the lifetime of the bond as the difference between the time when clamping force is attained and the time when the bond dissociates. This persistence time of the bond gives the bond lifetime. The mean lifetime of the bond at each clamping force is obtained by fitting the survival probabilities with exponential decay functions. An F-test was used to estimate the best fit and found that for all the experiments with *individual* tip-links (heterodimers), mono-exponential decay is the best fit over bi-exponential decay. In contrast, the heterotetrameric tip-link complexes are best fit to mono-exponential decays at low forces, while bi-exponential decays appeared as the best model in the high force-regime (>70 pN) (see Supplementary Table 1). The emergence of bi-exponential at high forces could be due to the failure in the rebinding, as reported previously by Wesley et al. *Nat. Comm*. 2021.

The data in Fig. 1f is fitted as follows:

1 $$\bullet\;{{{{{\rm{First}}}}}}\; {{{{{\rm{Slip}}}}}}\; {{{{{\rm{Region}}}}}}\!: < \, \tau \, > ={\tau }_{0}\exp \left(-\frac{f}{{f}^{*}}\right){with} \, {\tau }_{0}=31.8{{{{{\rm{;}}}}}}\,{f}^{*}=24.5$$

2 $$\bullet\;{{{{{\rm{Second}}}}}}\; {{{{{\rm{Slip}}}}}}\; {{{{{\rm{Region}}}}}}\!: < \, \tau \, > ={\tau }_{0}\exp \left(-\frac{f}{{f}^{*}}\right){with}\,{\tau }_{0}=8.4{{{{{\rm{;}}}}}}\,{f}^{*}=231.3$$

3 $$\bullet\;{{{{{\rm{Ideal}}}}}}\; {{{{{\rm{Region}}}}}}\!: < \, \tau \, > ={\tau }_{0}\,{with}\,{\tau }_{0}=6.3$$

- % error in fitting first slip region: 5.7 %
- % error in fitting ideal region: 0.4%
- % error in fitting second slip region: 5.5%

Further, the strikingly different values of the fitting parameter, *f* ^*^, for the two slip regions clearly indicate that a single-exponential decay cannot cover the entire force-lifetime feature.

### Sliding-rebinding model and its modification

In the sliding-rebinding model, the binding interface is formed by two identical pairs of pseudoatoms with population *P*_11_. This interface may dissociate in two competing pathways. Pathway one follows the conventional route where the interface disintegrates under tension to reach transient states with populations *P*_10_/*P*_01_, which can undergo complete dissociation to *P*_00_ (Supplementary Fig. 20). The force-induced dissociation-rates for the single-bond (*k*_-1_) and double bonds (*k*_-2_) are described using Bell’s model^50^ in Eq. 8, 9. The second route involves more steps. The tensile force reorients the interacting domains and instead of complete dissociation, facilitates the formation of new binding interface, ${P}_{10}^{{\prime} }$ and eventually ${P}_{11}^{{\prime} }$. The probability of forming new interactions, *P*_*n*_, which is defined in Eq. (10), depends on the tensile forces. Though the binding interfaces for ${P}_{10}^{{\prime} }$ and ${P}_{11}^{{\prime} }$ are new, kinetics of the bond-formation and dissociation are identical to the old ones. For the mutant tip-links, the loss of pivot prohibits rebinding, so we set ${P}_{n}=0$. We derived the rate-equations for the WT and mutant interfaces in Eqs. 4–8, 12–14. ${k}_{+1}\&{k}_{+2}$ in the rate-equations are considered as the basal or intrinsic rate-constants of reorganization and rebinding of bonds, respectively.

Following rate-equations are constructed for the WT tip-link interface:

4 $$\frac{d{P}_{11}}{{dt}}=2{k}_{+1}{P}_{10}+{k}_{+2}{P}_{01}-{k}_{-2}{P}_{11}$$

5 $$\frac{d{P}_{10}}{{dt}}={k}_{-2}{P}_{11}-2\left({k}_{+1}+{k}_{-1}\right){P}_{10}$$

6 $$\frac{d{P}_{01}}{{dt}}=2{P}_{n}{k}_{-1}{P}_{10}-\left({k}_{+2}+{k}_{-1}\right){P}_{01}$$

7 $$\frac{d{P}_{00}}{{dt}}=2({1-P}_{n}){k}_{-1}{P}_{10}+{{k}_{-1}P}_{01}$$

${k}_{-1} \,\&\, {k}_{-2}$ are the force-dependent dissociation-rates that follow the Bell equation^50^:

8 $${k}_{-1}\left(f\right)={k}_{-1}^{0}\times \exp \left(f\times \frac{{x}_{{{{{{\rm{\beta }}}}}}}}{{k}_{B}T}\right)$$

9 $${k}_{-2}\left(f\right)={2.k}_{-1}^{0}\times \exp \left(f\times \frac{{x}_{{\beta }}}{{2k}_{B}T}\right)$$

${k}_{-1}^{0}$ is the intrinsic off-rate at no-force and *x*_*β*_ is the distance from bound state to the transition state. Probability (*P*_*n*_) of making new bonds and undergoing stronger-binding is defined as follows:

10 $$\left.\begin{array}{cc}{P}_{n}=0 \hfill& \hfill 0 \, < \, {{{{{\rm{F}}}}}} \, < \, {{{{{{\rm{F}}}}}}}_{{{{{{\rm{C}}}}}}1} \\ {P}_{n}={\left\{0.5\left[1-\cos \left(\frac{\pi F}{{F}_{c2}}\right)\right]\right\}}^{n} & {{{{{{\rm{F}}}}}}}_{{{{{{\rm{C}}}}}}1} \, < \,=\, {{{{{\rm{F}}}}}} \, < \,=\, {{{{{{\rm{F}}}}}}}_{{{{{{\rm{C}}}}}}2} \\ {P}_{n}=1 \hfill& \hfill {{{{{\rm{F}}}}}} \, > \, {{{{{{\rm{F}}}}}}}_{{{{{{\rm{C}}}}}}2}\end{array}\right\}$$

Here, *n* is the fitting parameter, and ${F}_{C1} \,\&\, {F}_{C2}$ are the critical forces that define the first slip to catch transition and catch to second slip transition respectively. Probability rate equations were solved analytically using MATLAB and survival probability was estimated as

11 $$1-{P}_{00}={P}_{11}+{P}_{10}+{P}_{10}^{{\prime} }$$

Following rate-equations are constructed for the mutant tip-link interface:

12 $$\frac{d{P}_{11}}{{dt}}=2{k}_{+1}{P}_{10}-{k}_{-2}{P}_{11}$$

13 $$\frac{d{P}_{10}}{{dt}}={k}_{-2}{P}_{11}-2\left({k}_{+1}+{k}_{-1}\right){P}_{10}$$

14 $$\frac{d{P}_{00}}{{dt}}=2.{{k}_{-1}P}_{01}$$

### For unfolding associated unbinding kinetics of tip-links involving non-interacting domains

We calculated the force-dependent unfolding probabilities for the most predominant unfolding step heights ( ~ 5 and ~12 nm) by the following equations^51,52^:

15 $$\left.\begin{array}{c}{P}_{1u}=1-\exp (-{k}_{1u}t)\\ {P}_{2u}=1-\exp (-{k}_{2u}t)\end{array}\right\}$$

Where ${P}_{1u}$, and ${P}_{2u}$ are the unfolding probabilities of 5 nm and 12 nm, respectively. ${k}_{1u}$ and ${k}_{2u}$ are their corresponding force-dependent unfolding rates which are estimated from the Bell equation:

16 $$\left.\begin{array}{c}{k}_{1u}={k}_{1u}^{0}\times \exp (\;f.\,{x}_{\beta }^{1u}/{k}_{B}T)\\ {k}_{2u}={k}_{2u}^{0}\times \exp (\;f.\,{x}_{\beta }^{2u}/{k}_{B}T)\end{array}\right\}$$

To incorporate the unfolding from all the tip-link variants to the model, we segregated the lifetime-force data for all the step-heights ~5, ~12, ~22, ~34 nm for full-length Cdh23 EC1–27 and fitted to an exponential decay (Bell model) to obtain the zero-force unfolding rate (${k}_{u}^{0}$) and distance to transition-state (${x}_{\beta }^{u}$) for the individual step height, Fig. 3g and Table 4.

Further, we determined the unfolding probabilities ${P}_{1u}$ and ${P}_{2u}$ at each force using Eqs. 12, 13. The total unfolding probability is given as:

17 $${P}_{U}=\left({n}_{1N}\times {P}_{1u}+{n}_{2N}\times {P}_{2u}\right)$$

where

$${n}_{1N}={n}_{1}/({n}_{1}+{n}_{2})$$

$${n}_{2N}={n}_{2}/({n}_{1}+{n}_{2})$$

*n*_1_ and *n*_2_ are the force-dependent number of unfolding for 5 nm and 12 nm respectively, observed from the experimental data for all the tip-link variants.

Total unfolding-unbinding probability can be given as:

18 $${P}_{00u}={P}_{U}\times {P}_{00}$$

Finally, survival probability was determined as before, ${1-P}_{00u}$.

### Molecular dynamics simulations

We used the crystal structures of the complexes of EC12 domains of Cdh23 and Pcdh15 (PDB ID 4AQ8)^7^ and EC1-2 domains of Cdh23 and EC13 domains of Pcdh15 (PDB ID 6N2E)^19^ as the starting structure for MD simulations. For both the complexes, the simulation setup was made with Charmm-GUI^53,54^ using Charmm36 force field^55^ along with TIP3P water model^56^. This combination of force-field and water model has been shown to be appropriate for well-folded proteins (PMID: 29735687). The long axes of the complexes were aligned to the X-axis of the box. EC12 complex was immersed in a water box with dimensions 15 × 7.2 × 7.2 nm, while the EC12-EC13 complex was immersed in a box with dimensions 19 × 11 × 11 nm. For both systems, all box angles were set equal to 90°. The system was neutralized, and the final salt concentration was set to 0.15 M using sodium and chloride ions. The simulations were run using GROMACS 2020.4^57^ patched with PLUMED 2.7.4^58,59^. We used periodic boundary conditions and restrained the bonds involving hydrogen atoms using LINCS algorithm^60^. Particle Mesh Ewald method^61^ was used for calculating electrostatic interactions with a cutoff of 1.2 nm for long range interactions. The system was minimized for 10,000 steps using steepest descent algorithm followed by NVT equilibration for 5 ns with restraints on the protein backbone. The equilibration part of the trajectories was omitted from any analysis. This was followed by three independent FISST (Infinite Switch Simulated Tempering in Force)^31^ simulations of 200 ns each under the NPT ensemble. We used the X-component of distance between the C-terminal residues of Cdh23 and Pcdh15 as the collective variable for EC12 complex simulations. We used a force range of 10 to 150 pN with 61 interpolated points. The temperature was maintained at 303.15 K using a Nose-Hoover thermostat and for NPT simulations, the pressure was maintained at 1 bar using Parrinello-Rahman barostat.

The end-to-end distance was calculated using PLUMED and the buried surface area was calculated using the sasa module of VMD. The weighted histograms for distance and buried surface area were generated using the scripts available with the PLUMED-NEST entry (plumID:20.017). The frames corresponding to different force ranges were separated using python scripts employing MDAnalysis^62^. The inter-protein interactions including salt-bridges, hydrogen bonds, and hydrophobic interactions were calculated using PyInteraph^63^. The pairwise residue forces were calculated using gromacs-fda^64^ (release 2020) and written as signed scalars calculated by taking the norm of the force vector with the sign of the force determined based on the cosine of the angle between the force vector and vector joining the centers of masses of the two residues.

### Langevin dynamics simulations with the semiflexible filaments

We model a semiflexible filament as made up of N beads, where consecutive beads at positions *r*_*i*_ interact via the stretching energy

$${\in }_{s}=\mathop{\sum }\limits_{i=1}^{N-1}\frac{A}{2\sigma }{\left[{\vec{b}}_{i}-\sigma {\hat{t}}_{i}\right]}^{2},$$

characterized by the stiffness *A* and bond-length *σ*. The spring constant, ${k}_{{sp}}=\frac{A}{\sigma }$. The ith bond ${\vec{b}}_{i}$ = ${\vec{r}}_{i+1}$ − ${\vec{r}}_{i}$ is oriented along the local tangent vector ${\hat{t}}_{i}={\vec{b}}_{i}/| {\vec{b}}_{i}|$. The bending rigidity cost is given as^65^,

$${\in }_{b}=\mathop{\sum }\limits_{i=1}^{N-2}\frac{\kappa }{2\sigma }{\left[{\hat{t}}_{i+1}-{\hat{t}}_{i}\right]}^{2}$$

where κ is the bending rigidity.

The self-avoidance of the filament is implemented through a short-ranged Weeks–Chandler–Anderson (WCA) repulsion between all the non-bonded pairs of beads i and j^66^,

$${\in }_{{WCA}}	=4\in \left[\left({\frac{\sigma }{{r}_{{ij}}}}\right)^{12}-\left({\frac{\sigma }{{r}_{{ij}}}}\right)^{6}+\frac{1}{4}\right],\, {if} \, {r}_{{ij}} \, < \, {2}^{\frac{1}{6}}\,\sigma \\ 	=0,\,{{{{{\rm{otherwise}}}}}}$$

Thus, the full polymer model is described by the energy cost $\in={\in }_{s}+{\in }_{b}+{\in }_{{WCA}}$. Bonds between two semiflexible filaments are modelled via elastic springs. The extension ∆*r* of the bond between the monomers of the two filaments generates a load ${f}_{i}=-{k}_{m}\Delta r$ with *k*_*m*_ being the stiffness of the spring.

We consider two arrangements of semiflexible filaments with elastic interactions between them as follows:

1. In one arrangement which we call a dimeric configuration, we consider two filaments with partial bonding between them as shown in Fig. 4a. One end of the filament representing Pcdh15 (red) is tethered while a constant external force *F*_*e*_ is applied to one end of the filament. This external force can lead to the unbinding of the filaments with the bond detachment rate *ω*_*off*_. The detachment rate of individual bonds between filaments is modelled as a catch-slip bond with the rate given as^39^

$${\omega }_{{off}}={\omega }_{0}^{(1)}\exp \left(\frac{{f}_{1}}{{f}_{d}^{(1)}}\right)+{\omega }_{0}^{(2)}\exp \left(-\frac{{f}_{1}}{{f}_{d}^{(2)}}\right)$$

Where ${\omega }_{0}^{(1)}$, ${\omega }_{0}^{(2)}$ are bare detachment rates, *f*_1_ = | *f*_1_ | and ${f}_{d}^{(1)}$ and ${f}_{d}^{(2)}$ set the scales of the detachment forces. This choice of *ω*_*off*_ leads to a slip-catch-slip behavior as shown in Fig. 4a.

2. In the second arrangement, we consider a double dimeric arrangement leading to a tetrameric configuration consisting of four semiflexible filaments as shown in Fig. 4b. Filaments which are not part of the same dimeric configuration can also bind via elastic springs with spring constant *k*_*m*_. The external force now acts simultaneously on the ends of the two filaments as shown.

We perform molecular dynamics simulations of the bead spring polymers with beads of unit mass m = 1, in the presence of a Langevin heat bath. The bath fixes the ambient temperature ${k}_{B}T$ through a Gaussian white noise which obeys $\langle {\eta }_{i}(t)=0\rangle {and}\langle {\eta }_{i}(t){\eta }_{j}({t}^{{\prime} })\rangle=2\alpha {K}_{B}T{\delta }_{{ij}}\delta (t-{t}^{{\prime} }),$ with *α* denoting the viscosity of the environment. We express energy in units of ${k}_{B}T=\frac{1}{\beta }$ lengths in units of *σ* and time in units of $\tau=\alpha {\sigma }^{2}/{k}_{B}T=\beta \alpha {\sigma }^{2}$ A time step of $\delta t=0.001\tau$ is used for the Langevin dynamics. We present results with averages performed over 100 initial conditions.

We use a bond stiffness of $A=5\times {10}^{3}{k}_{B}T$/*σ* for a filament of *N* = 35 beads which ensures smaller bond fluctuations. For an equilibrium worm like chain, the ratio of the contour length $L=(N-1)\sigma$ to the persistence length $\lambda=\frac{2\kappa }{[(d-1){k}_{B}T]}$ given by the rigidity parameter $u=\frac{L}{\lambda }$, determines the semiflexibility of the chain with *d* as the dimension. In two dimensions, $\lambda=\frac{2\kappa }{{k}_{B}T}$. The end-to-end distribution of such a filament behaves like a completely flexible polymer with a single peak at zero separation at *u* ≈ 10 and a rigid-rod behavior with a single peak near full extension at *u* ≈ 1. We choose *u* = 3.33 where the free energy is known to show a characteristic double minimum corresponding to the co-existence of both rigid rod and flexible chain behaviors. This corresponds to $\kappa \beta /\sigma=5.1$ in the reduced units. We use ${k}_{m}=3\times {10}^{-3}\frac{A}{\sigma }$ and ${f}_{d}^{(1)}=\frac{5{k}_{B}T}{\sigma }$ and ${f}_{d}^{(2)}=\frac{10{k}_{B}T}{\sigma }$. The dimensionless bare detachment rates used are ${\omega }_{0}^{(1)}\tau=4\times {10}^{-6}$ and ${\omega }_{0}^{(2)}\tau=0.1.$

In real units, we choose the bond length $\sigma=10{nm}=0.01\mu m.$ At ambient temperature, ${k}_{B}T=0.0042{pN}\mu m.$ This leads to a force unit $\frac{{k}_{B}T}{\sigma }=0.42{pN}$. In a viscous environment with $\gamma=1{pN}-s/{\mu m}^{2}$, the viscous drag on a bond can be calculated as $\alpha=3\pi \gamma \sigma \approx 0.1{pNs}/\mu m.$ This sets the time scale $\tau=\frac{\alpha {\sigma }^{2}}{{k}_{B}T}\approx 2.38\times {10}^{-3}s.$ Bending rigidity *κ*=5.1${k}_{B}T\sigma \approx 2.142\times {10}^{-4}{pN}-{\mu m}^{2}$. $A=5\times {10}^{3}{k}_{B}T$/$\sigma \approx$ $2.1\times {10}^{3}{pN}$ and stiffness of the elastic bonds between filaments ${k}_{m}=3\times {10}^{-3}\frac{A}{\sigma }\approx 0.63\frac{{pN}}{{nm}}$. The values of the detachment forces are ${f}_{d}^{(1)}=2.2{pN}$; ${f}_{d}^{(2)}=4.2{pN}$ and those of the bare detachment rates are ${\omega }_{0}^{(1)}\approx 1.68\times {10}^{-3}{s}^{-1}$ and ${\omega }_{0}^{(2)}\approx 42{s}^{-1}$ respectively.

### Reporting summary

Further information on research design is available in the Nature Portfolio Reporting Summary linked to this article.

### Supplementary information

Supplementary Information

Description of additional supplementary files

Supplementary Movie 1

Supplementary Movie 2

Reporting Summary

### Source data

Source Data

## Data Availability

All data are available in manuscript, supplementary file and source data file. Source data are provided with this paper.

## Code availability

All home-written MATLAB codes used here are deposited in the GitHub repository https://github.com/Sabyasachi-Rakshit/Arora-et.-al-Nature-Comm.git.
