# Supplementary material for: Emergence of slip-ideal-slip behavior in tip-links serve as force filters of sound in hearing
Source: Nat Commun. 2024 Feb 21;15:1595. doi: 10.1038/s41467-024-45423-8 (PMC10881517; doi:10.1038/s41467-024-45423-8)
Supplement: Supplementary file 1 — Supplementary Information [file 41467_2024_45423_MOESM1_ESM.pdf]

**Supplementary Information for**

# Emergence of slip-ideal-slip behavior in tip-links serve as force filters of sound in hearing

**Nisha Arora[1#], Jagadish P. Hazra[1#], Sandip Roy[2], Gaurav K. Bhati[1], Sarika Gupta[3], K. P. Yogendran[2], Abhishek Chaudhuri[2*], Amin Sagar[4*], and Sabyasachi Rakshit[1*]**

[1]Department of Chemical Sciences, Indian Institute of Science Education and Research Mohali, Punjab, India.

[2]Department of Physical Sciences, Indian Institute of Science Education and Research Mohali, Punjab, India.

[3]National Institute of Immunology, New Delhi, India

[4]Centre de Biochimie Structurale, INSERM, CNRS, Université de Montpellier, Montpellier, France

[*]Correspondence to:

Email: srakshit@iisermohali.ac.in; abhishek@iisermohali.ac.in; amin.sagar@cbs.cnrs.fr

[#] Equal contributions

**Supplementary Text** **4**

Dynamic force balance protects essential interactions in the Cdh23-Pcdh15 interface

**Supplementary Figures and Legends** **6**

Supplementary Figure 1: Nomenclature used in this study for the tip-link 6

Supplementary Figure 2: SDS-PAGE confirmed the dimerization of the cadherin proteins in the solution 7

Supplementary Figure 3: Exponential decay fitting of survival plots for tip-link *complex* 8

Supplementary Figure 4: Resulting amplitudes A1, and A2 and lifetimes from double-exponential fitting of the survival plots 9

Supplementary Figure 5: Unfolding step height distribution for tip-link *complex* 10

Supplementary Figure 6: Instantaneous unfolding for tip-link *complex* 11

Supplementary Figure 7: The binding energy between the two proteins increases with application of force 12

Supplementary Figure 8: Hydrogen bond survival with force obtained from FISST simulations 13

Supplementary Figure 9: Survival of hydrophobic interactions with force obtained from FISST simulations 14

Supplementary Figure 10: Force-dependent load balancing in Cdh23-Pcdh15 complex 15

Supplementary Figure 11: Correlation coefficient between the inter-residue forces for all the intra-protein residue pairs 16

Supplementary Figure 12: Exponential decay fitting of survival plots for Cdh23 EC1-5-Pcdh15 EC1-2 17

Supplementary Figure 13: Exponential decay fitting of survival plots for Cdh23 EC1-10-Pcdh15 EC1-2 18

Supplementary Figure 14: Exponential decay fitting of survival plots for Cdh23 EC1-21-Pcdh15 EC1-2 19

Supplementary Figure 15: Exponential decay fitting of survival plots for Cdh23 EC1-27-Pcdh15 EC1-2 20

Supplementary Figure 16: Unfolding prior to unbinding prolongs the bond lifetime 21

Supplementary Figure 17: Percentage of unfolding events in different tip-links variants 22

Supplementary Figure 18: Force-lifetime data for four major extensions from Cdh23 EC1-27-Pcdh15 EC1-2 clamp measurements 23

Supplementary Figure 19: Force-dependent lifetime behavior of semiflexible filaments linked with only slip-bonds 24

Supplementary Figure 20: Dissociation paths for the wild-type tip-link interface 25

Supplementary Table 1: F-statistical analysis for tip-link *complex* 26

## Supplementary Text

### Dynamic force balance protects essential interactions in the Cdh23-Pcdh15 *interface*

The salt-bridge interaction between Pcdh15(R113) and Cdh23(E78) remains unaltered in the entire clamping forces. We propose the pivotal role of this salt-bridge on the slip-to-catch switch under tension. To understand the *interface* wide bond-mechanics that conserve the pivotal role of this salt-bridge interaction at a large range of tension, we quantified how force is stored and transmitted in the tip-link *interface* consisting of Cdh23 EC1-2 and Pcdh15 EC1-2. The pairwise inter-residue force distributions **(Supplementary Figure 10a)** were calculated by using gromacs-fda(*54*) (release 2020) and written as signed scalars calculated by taking the norm of the force vector with the sign of the force determined based on the cosine of the angle between the force vector and vector joining the centers of masses of the two residues. Notably, proteins can be considered as pre-stressed molecules due to their local frustration, where the balance between the tensile and compressive forces determines the overall mechanical stability.

At the clamping force of 10-20 pN, which are relatively small forces for this *interface*, we measured tensed bond among the most important interprotein salt-bridge, i.e., Cdh23(E78) – Pcdh15(R113). Tensed bonds across proteomes tend to pull the proteins closer. Interestingly, the salt-bridge between Cdh23(E78) – Pcdh15(R113) maintains a constant degree of tension across the entire loading force **(Supplementary Figure 10b)**. A bond bearing a constant tension across large tensile forces refers to a load-balancing activity inside the protein complex that drives the force away from this critical interaction. In order to decipher the inter-residue interactions that might absorb the applied force and steer it away from these critical salt-bridges, especially from E78-R113, we calculated the correlation of pairwise inter-residue forces ($r_{af}^{f}$) with the applied force **(Supplementary Figure 10c)**. The residue pairs with $|r_{af}^{f}|$ significantly greater than zero, experience a change in the inter-residue forces as a function of applied force. These residue pairs should be responsible for actively distributing the load inside the proteins and the *interface,* thus assisting Cdh23(E78) – Pcdh15(R113) to remain unperturbed. On the other hand, residue pairs with $r_{af}^{f} \approx 0$ bear a consistent load irrespective of the applied force. Using the scale of $|r_{af}^{f}|$, we mapped the correlations among the load balancing residues **(Supplementary Figure 10c)** and identified crucial interactions between the EC1-EC1 and EC1-EC2 domains of Cdh23 and Pcdh15, respectively. Additionally, to highlight the residue-pairs that are strongly correlated to the applied force, we mapped the

differential inter-residue force (inter-residue force at a force range – inter-residue force at 10-20 pN) **(Supplementary Figure 10d)** and observed a strongly interconnected network in certain regions of the proteins (e.g., residues 150-175 and 100-125 for Pcdh15 and 75-100 and 130-150 for Cdh23) (black dashed lines, **Supplementary Figure 10c**). This network of interconnected residue pairs bears and distributes the applied force to maintain the integrity of the *interface*. For reference, we also mapped the correlation among load-bearing residues within the protein **(Supplementary Figure 11)** which implies that load balancing is not only happening at the interface but also within the protein.

**Supplementary Figures and Legends**

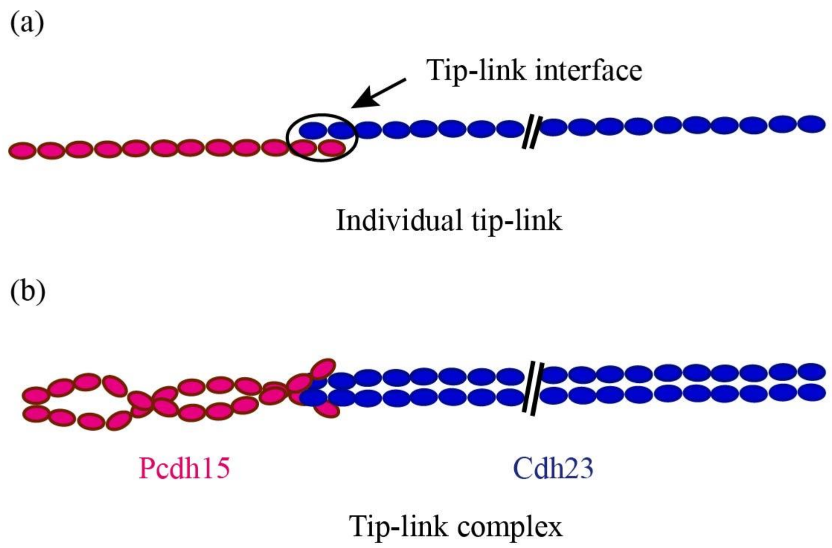


**Supplementary Figure 1: Nomenclature used in this study for tip-links (in support of figure 1 and 2 in main text). (a)** Tip-link constituted by monomeric Pcdh15 and Cdh23 is named as individual tip-link and the selected region here indicates the tip-link binding interface constituted by two outermost domains of both the proteins. **(b)** Tip-link made up of cis-dimers of Pcdh15 and a pair of Cdh23 is referred to as tip-link *complex*.

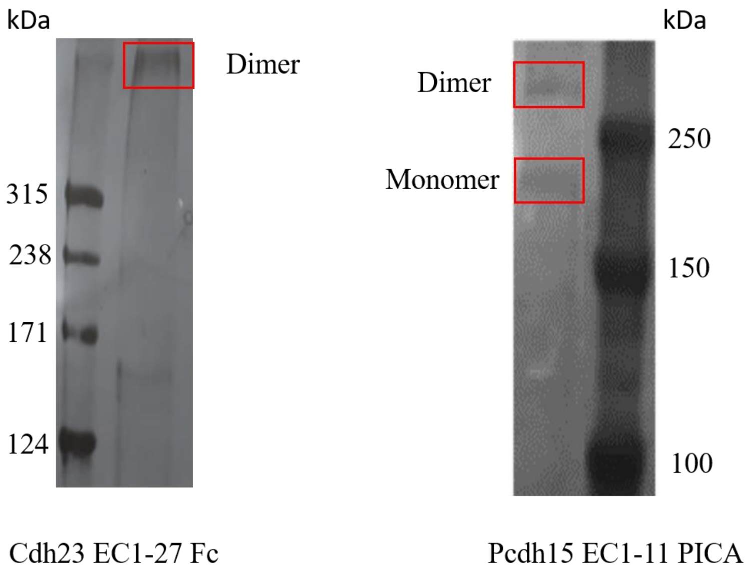


**Supplementary Figure 2. SDS-PAGE followed by silver-staining confirmed the dimerization of the individual tip-link cadherin proteins in the solution (in support of figure 1).** The left panel shows the silver-stained gel for Cdh23 EC1-27 Fc protein. Band appeared at a higher molecular weight corresponding to the dimer. The right panel shows the monomer and dimer bands of Pcdh15 EC1-11 PICA in a silver-stained SDS-PAGE gel. This experiment is replicated 3 times.

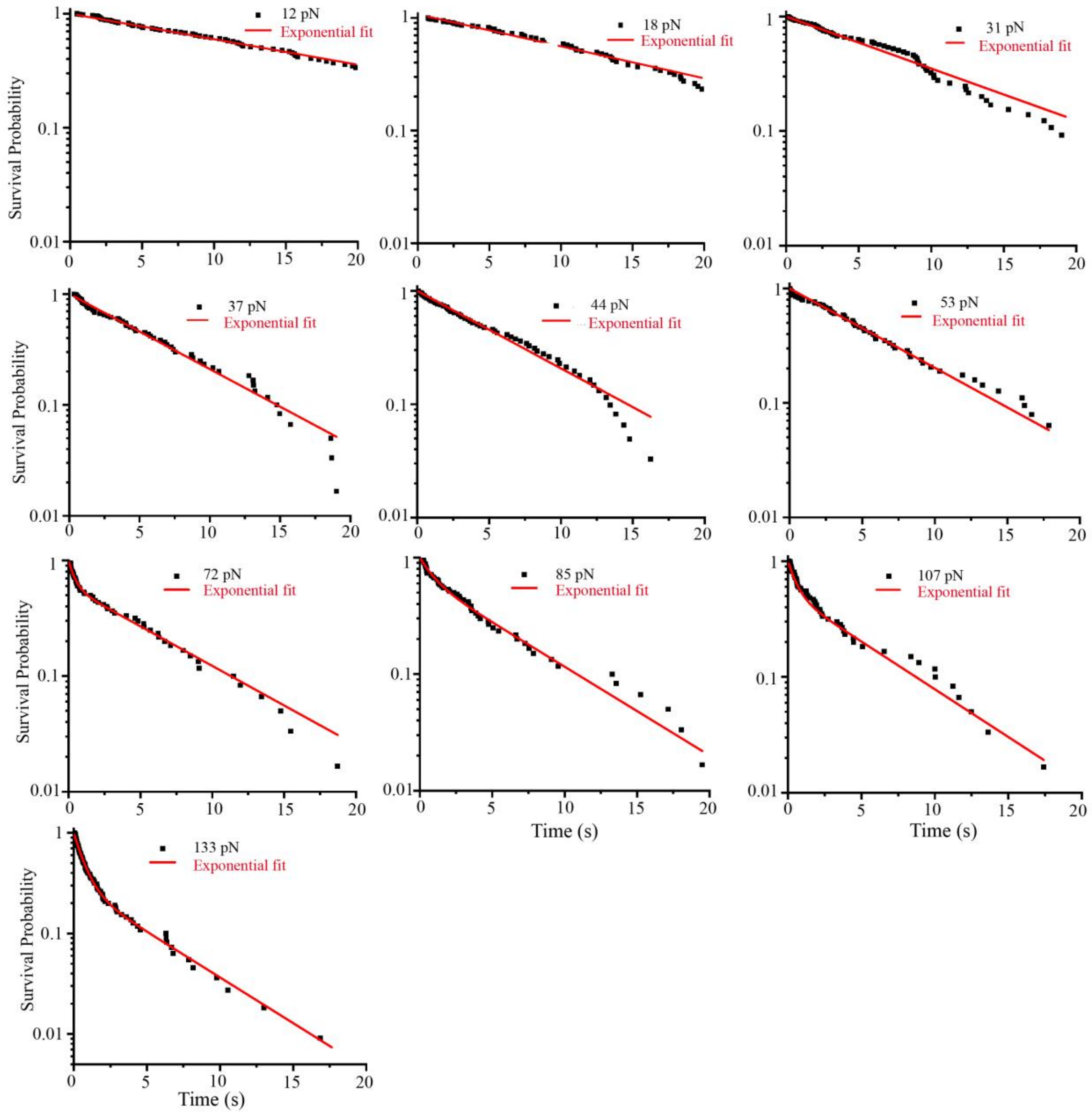


**Supplementary Figure 3. Exponential decay fitting of survival plots for tip-link *complex* (Cdh23 EC1-27 Fc-Pcdh15 EC1-11 PICA) at different clamping forces (in support of figure 1).** For tip-link *complex*, exponential decay fitting of the survival plot is shown separately at each clamping force. The survival probabilities were derived from n = 86, 73, 65, 62, 61, 63, 60, 60, 60, and 111 data points for the clamping forces of 12, 18, 31, 37, 44, 53, 72, 85, 107, and 133 pN, respectively. Survival plots at low forces from 12 pN to 53 pN are fitted with mono-exponential decay while higher forces from 72 pN to 133 pN are fitted with bi-exponential decay. The fitting models were selected from the one-sided F-tests with 99% confidence interval.

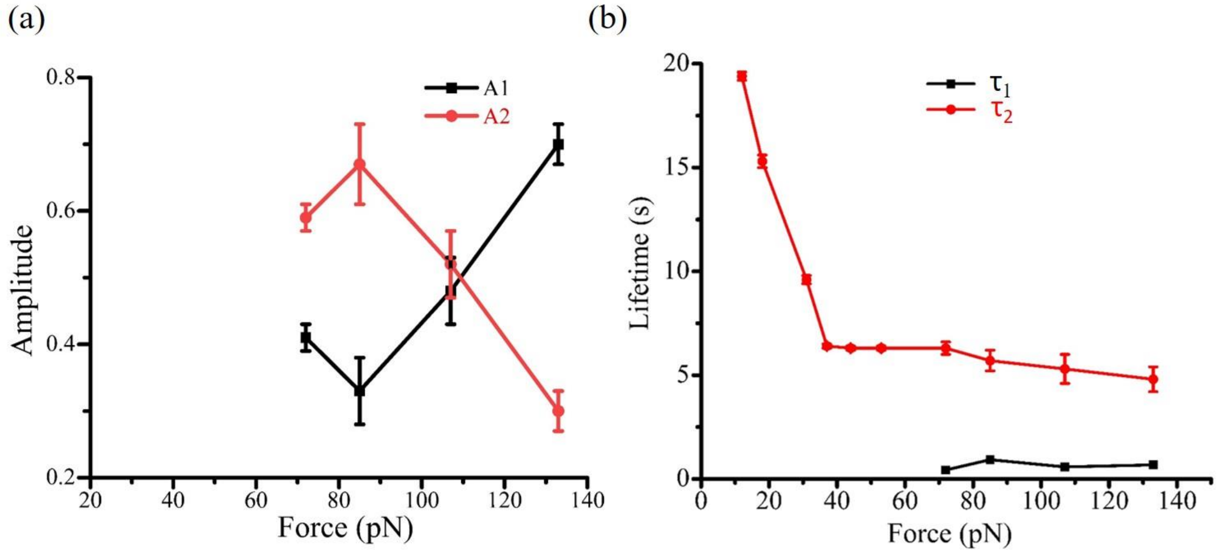


**Supplementary Figure 4. The survival probabilities of tip-link *complex* at higher forces (>70 pN) follow double-exponential decay (in support of figure 1).** The corresponding amplitudes, $A_1$, and $A_2$ (a) and lifetimes $\tau_1$, $\tau_2$ (b) from the double-exponential fitting of the survival plots are plotted here. Amplitude corresponding to the higher-lifetime component (red) decreases with force whereas amplitude corresponding to the lower lifetime component (black) increases with force. Intuitively, the short-lived component is arising from the dissociations of tip-links without re-binding. This is also reflected in the corresponding amplitude $A_1$, which increases with the force. Errors are the standard errors obtained from the exponential fitting of the survival probability curves.

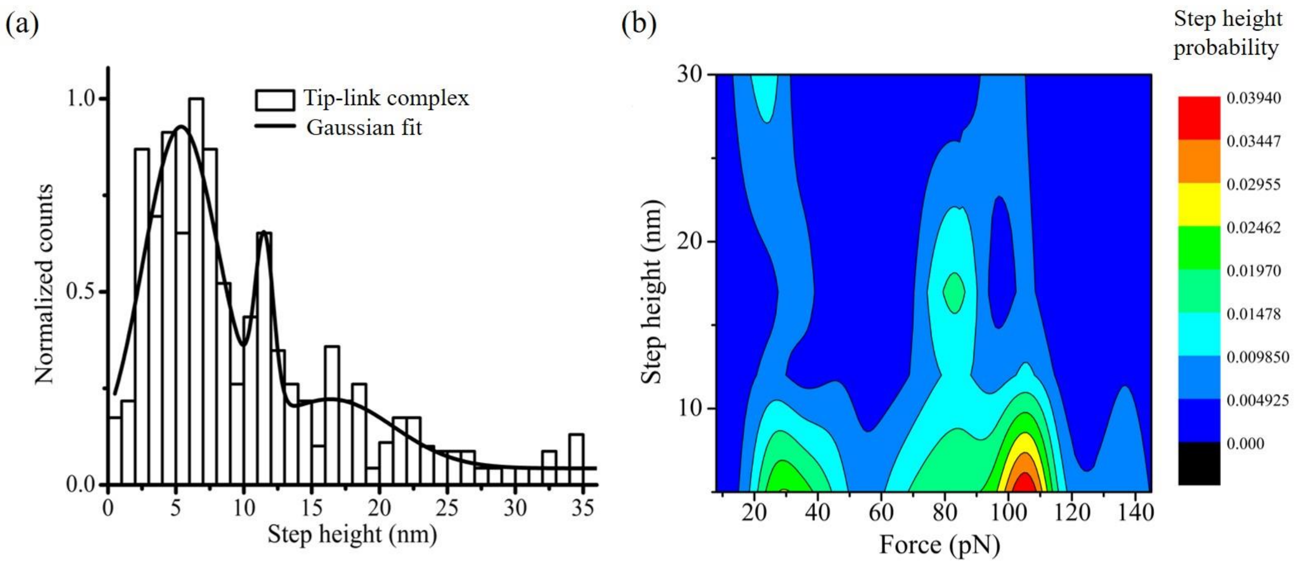


**Supplementary Figure 5. Unfolding step-height distribution of tip-link *complex* (in support of figure 1). (a)** Gaussian fitting of step height distribution (n = 241) resulted in three major peaks at 5.3±0.3, 11.5±0.2, and 16.4±2.8 nm. **(b)** Contour plot depicting the occurrence probability of the step-heights with force.

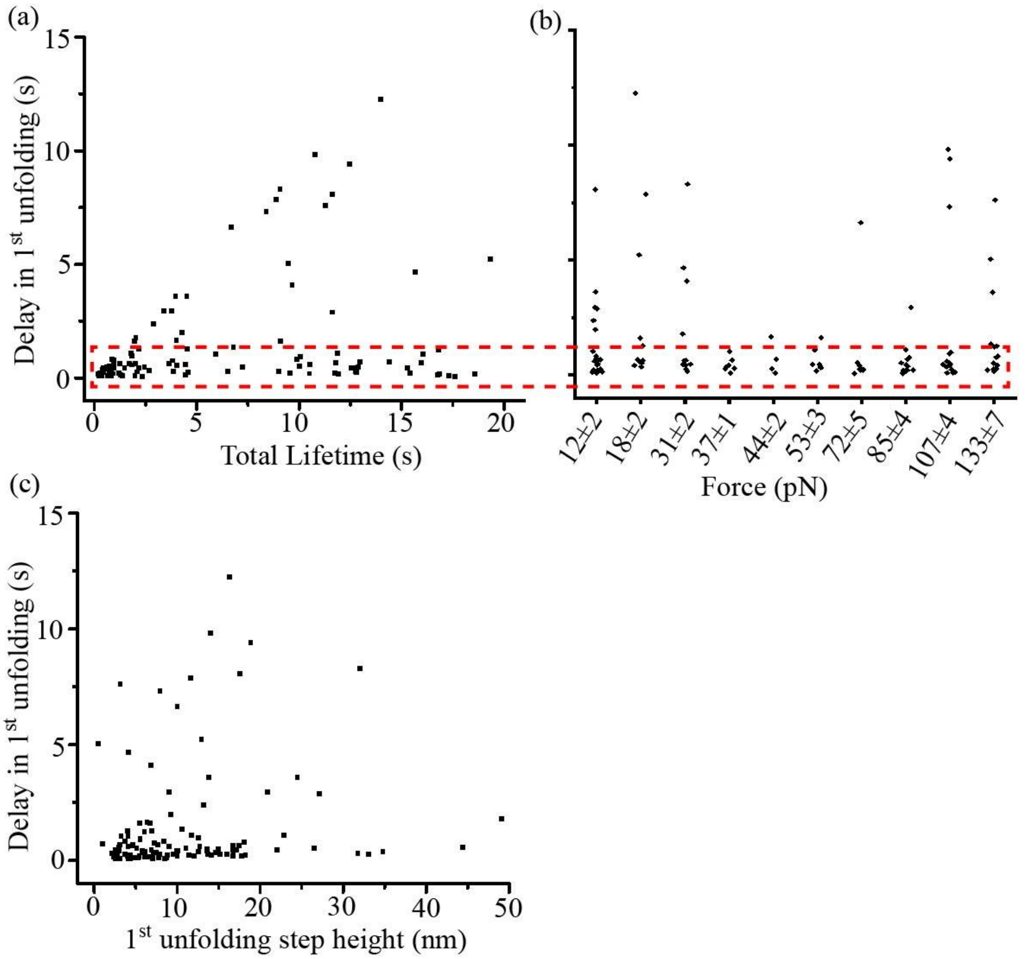


**Supplementary Figure 6: Instantaneous unfolding observed for heterotetramer tip-link *complex* (in support of figure 1). (a and b)** Delay in first unfolding after clamp was plotted with total lifetime of the tip-link *complex* (a) and with the clamping forces (b) for all the events where unfolding precedes the unbinding (n = 114). We observed that for most of the events (80 %), this delay in unfolding after the clamp clustered between 0.06 s - 1.35 s as shown by a dotted rectangular box. **(c)** Variation of delay in first unfolding with the corresponding unfolding step height. The number of data points (n) are same as (a).

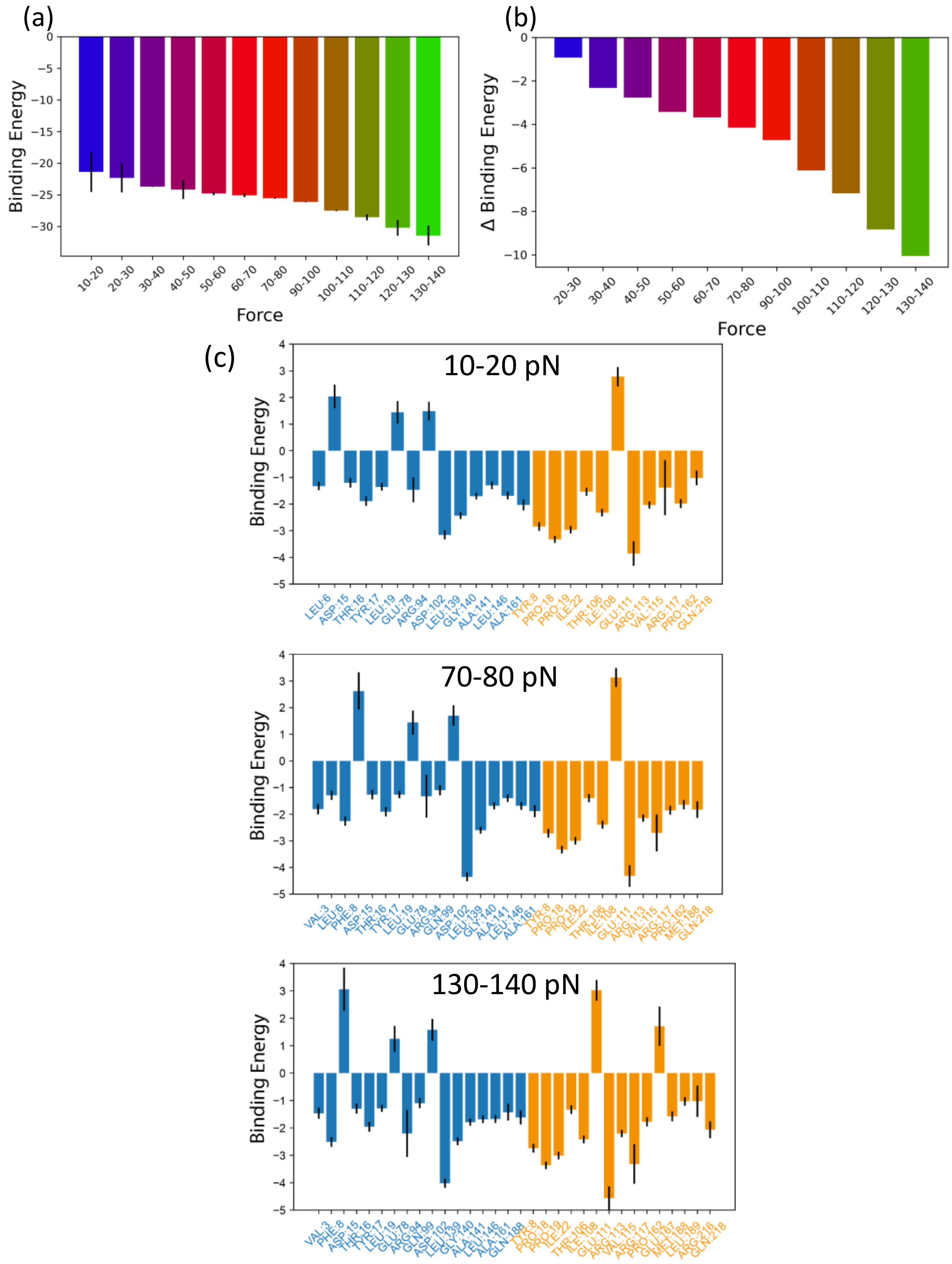


**Supplementary Figure 7: The binding energy between the two proteins increases with application of force (in support of figure 2). (a)** The binding energy between the two proteins calculated using MM-GBSA approach. **(b)** A plot showing the difference in the binding energy relative to the lowest force range to emphasize the increase in binding energy. **(c)** The per-residue decomposition of binding energy at three force ranges (10-20, 70-80 and 130-140 pN) (n=3 independent simulations, error bars show the standard deviation for all plots).

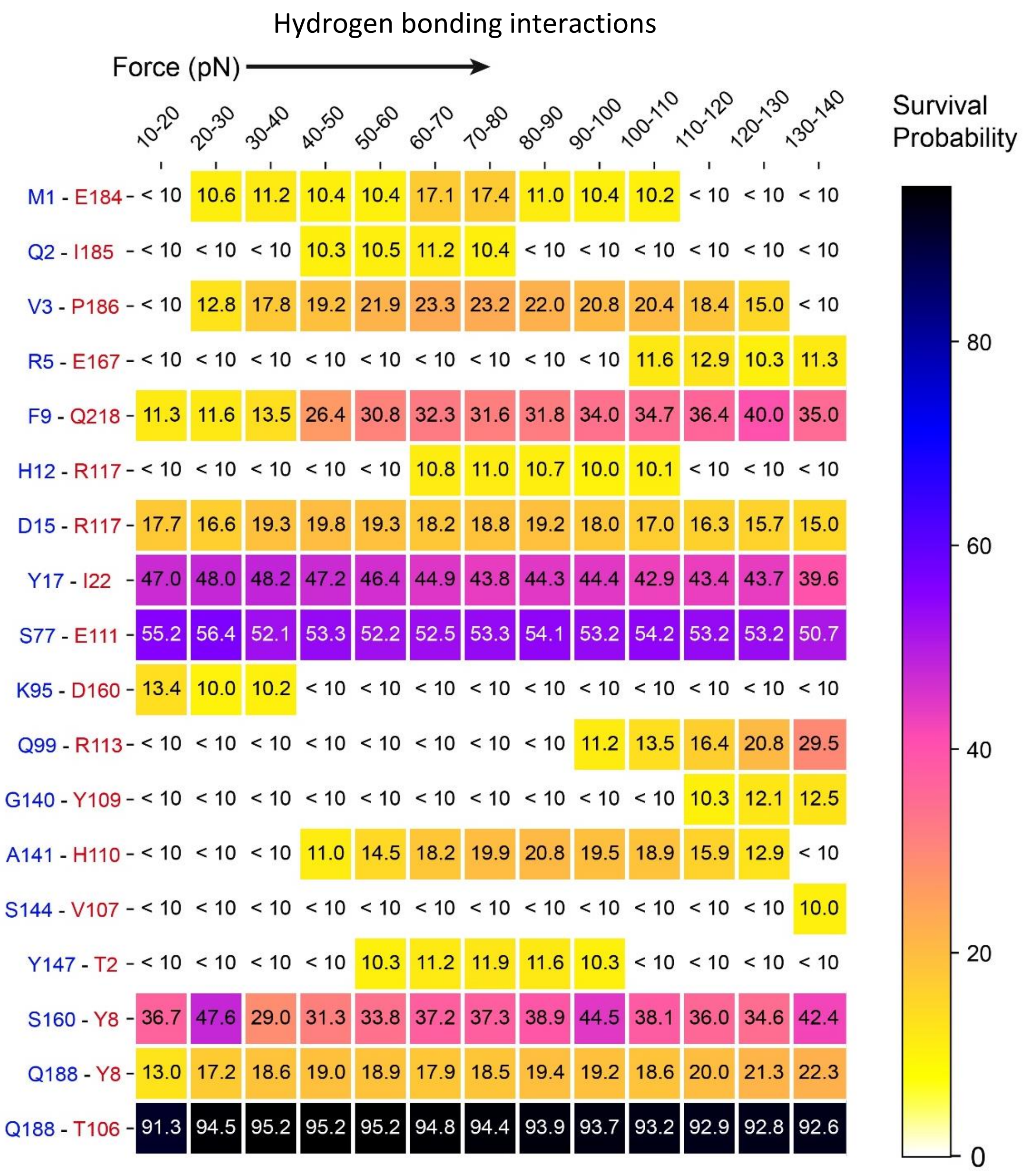


**Supplementary Figure 8: Hydrogen bond survival with force obtained from FISST simulations (in support of figure 2).** The percentage of frames showing the existence of specified hydrogen-bond interactions at different force ranges. The residue pair on the left depicts the hydrogen bond between residues of Cdh23 (blue) and Pcdh15 (red). (n=3 independent simulations)

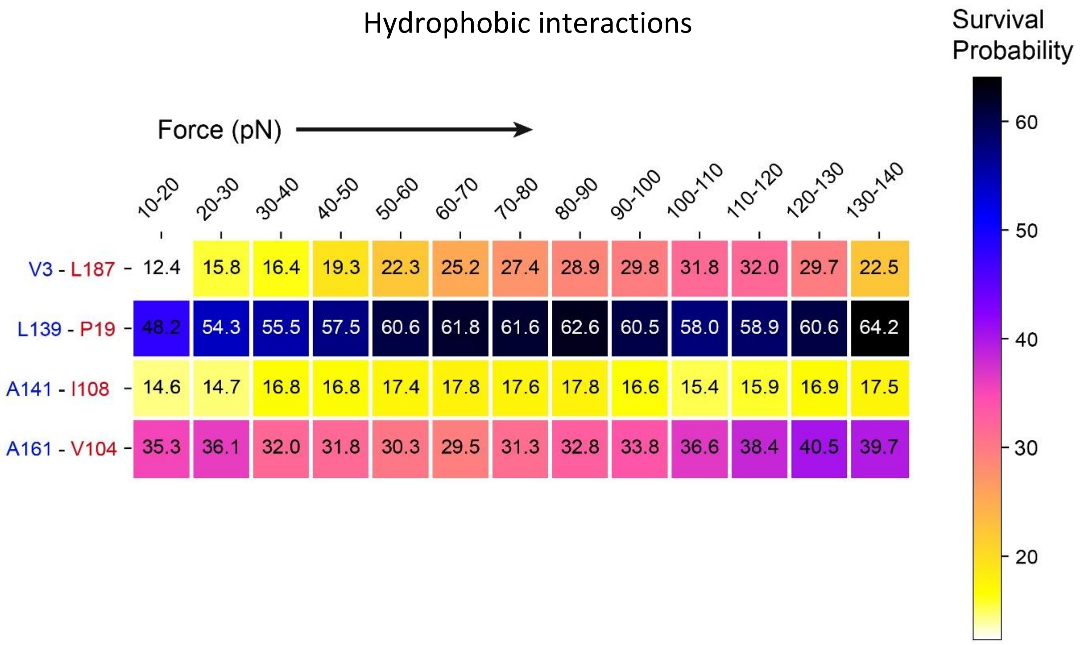


**Supplementary Figure 9: Survival of hydrophobic interactions with force obtained from FISST simulations (in support of figure 2).** The percentage of frames showing the existence of specified hydrophobic interactions at different force ranges. The residue pair on the left depicts the hydrophobic interaction between residues of Cdh23 (blue) and Pcdh15 (red). (n=3 independent simulations)

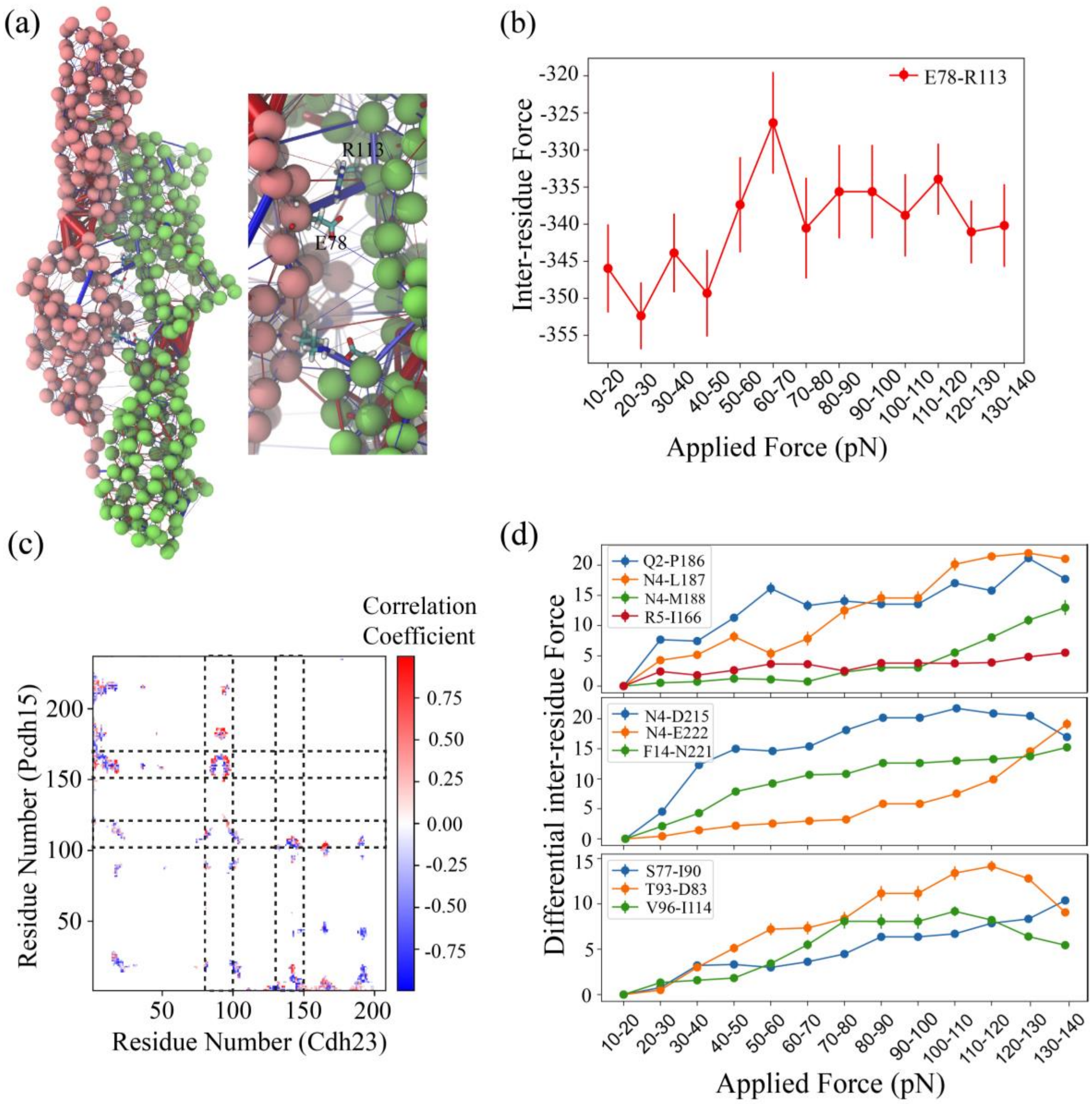


**Supplementary Figure 10: Force-dependent load balancing in Cdh23-Pcdh15 *interface* (in support of figure 2). (a)** The pairwise inter-residue forces plotted over the structure of Cdh23-Pcdh15 complex. The colour of the cylinders represents the sign of the force with blue being negative and red being positive. The width of the cylinders represents the magnitude of the force. **(b)** The inter-residue forces for the salt-bridge paired residues E78-R113. **(c)** A plot showing the correlation coefficient between the inter-residue forces and the applied force ($r_{af}^{f}$) for all the inter-protein residue pairs. **(d)** Some examples of residue pairs for which the inter-residue force is highly correlated with applied force ($r_{af}^{f}$ > 0.7) (n=3 independent simulation, error bars show the standard deviation). (n=3 independent simulations)

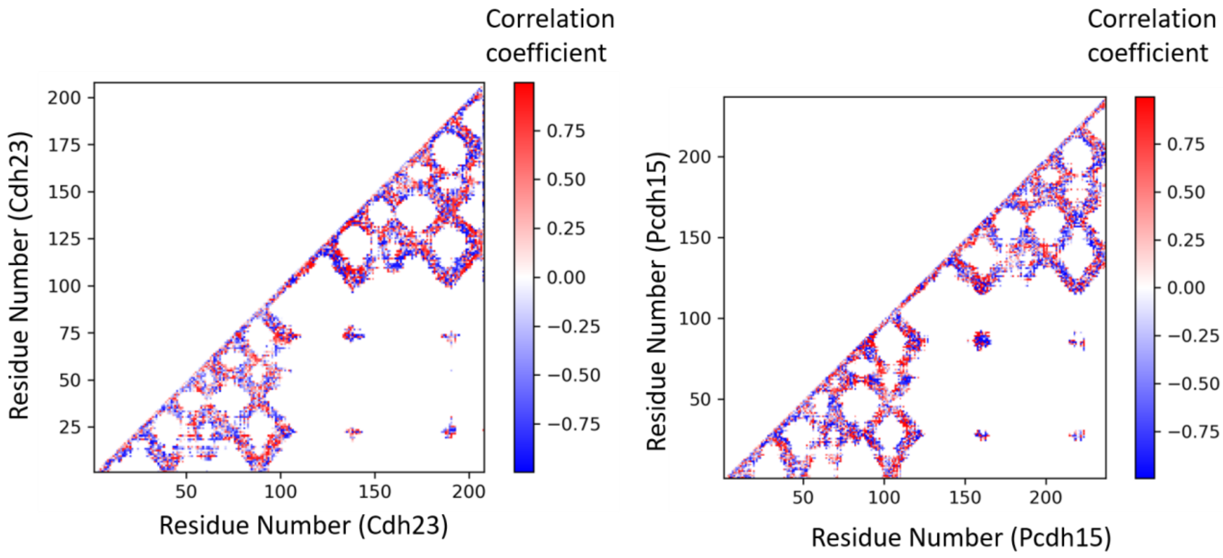


**Supplementary Figure 11: Correlation coefficient between the inter-residue forces and the applied force ($r^f_{af}$) for all the intra-protein residue pairs of Cdh23 and Pcdh15 EC1-2 (in support of figure 2).** (n=3 independent simulations)

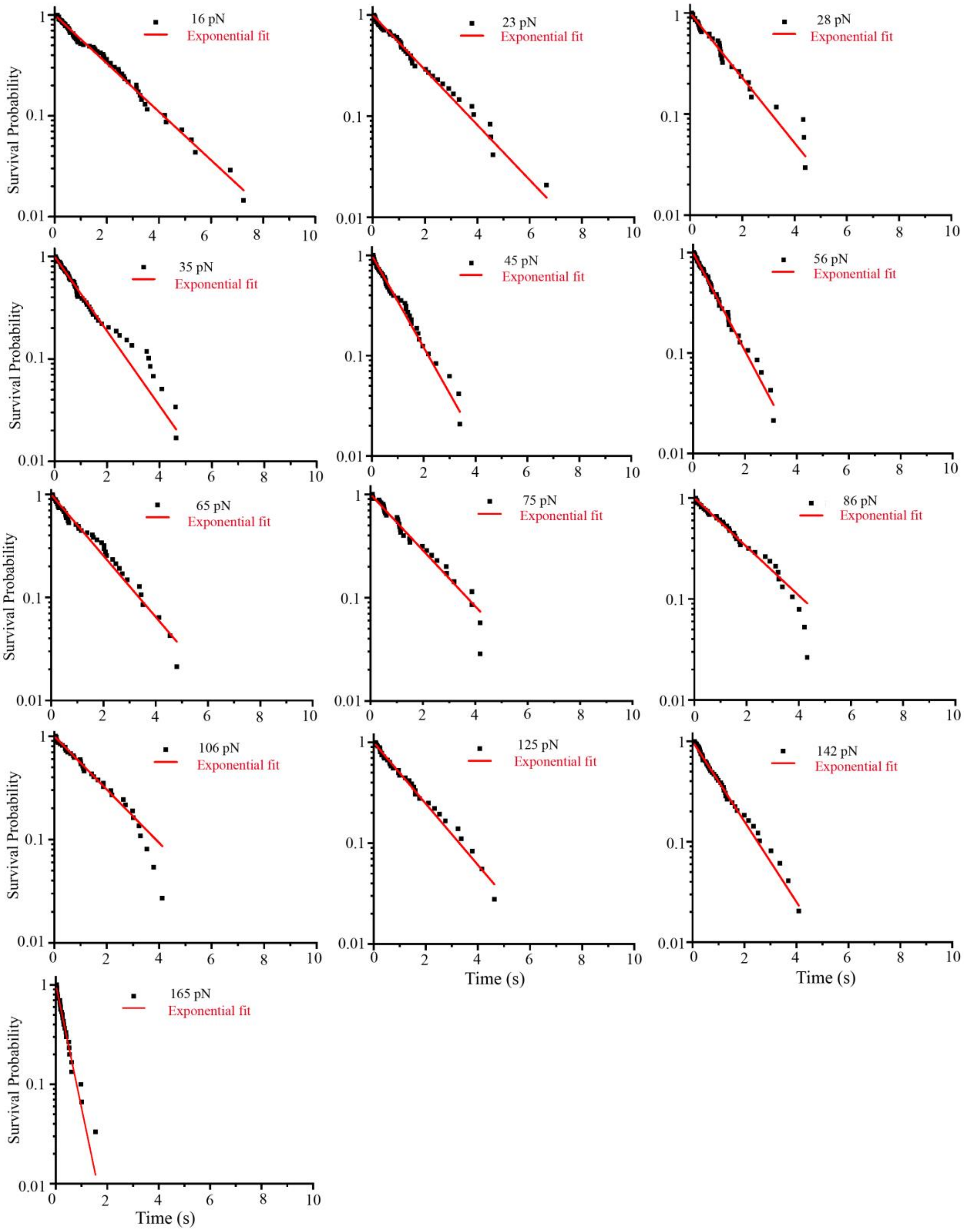


**Supplementary Figure 12: Exponential decay fitting of survival plot for *individual* tip-link comprising Cdh23 EC1-5 and Pcdh15 EC1-2 at different clamping forces (in support of figure 3).** For Cdh23 EC1-5-Pcdh15 EC1-2, mono-exponential decay fitting of the survival plot is shown separately at each clamping force. The survival probabilities were derived from n = 69, 48, 34, 59, 48, 47, 47, 35, 38, 37, 36, 49, and 30 data points for the clamping forces of 16, 23, 28, 35, 45, 56, 65, 76, 86, 106, 125, 142, and 165pN, respectively.

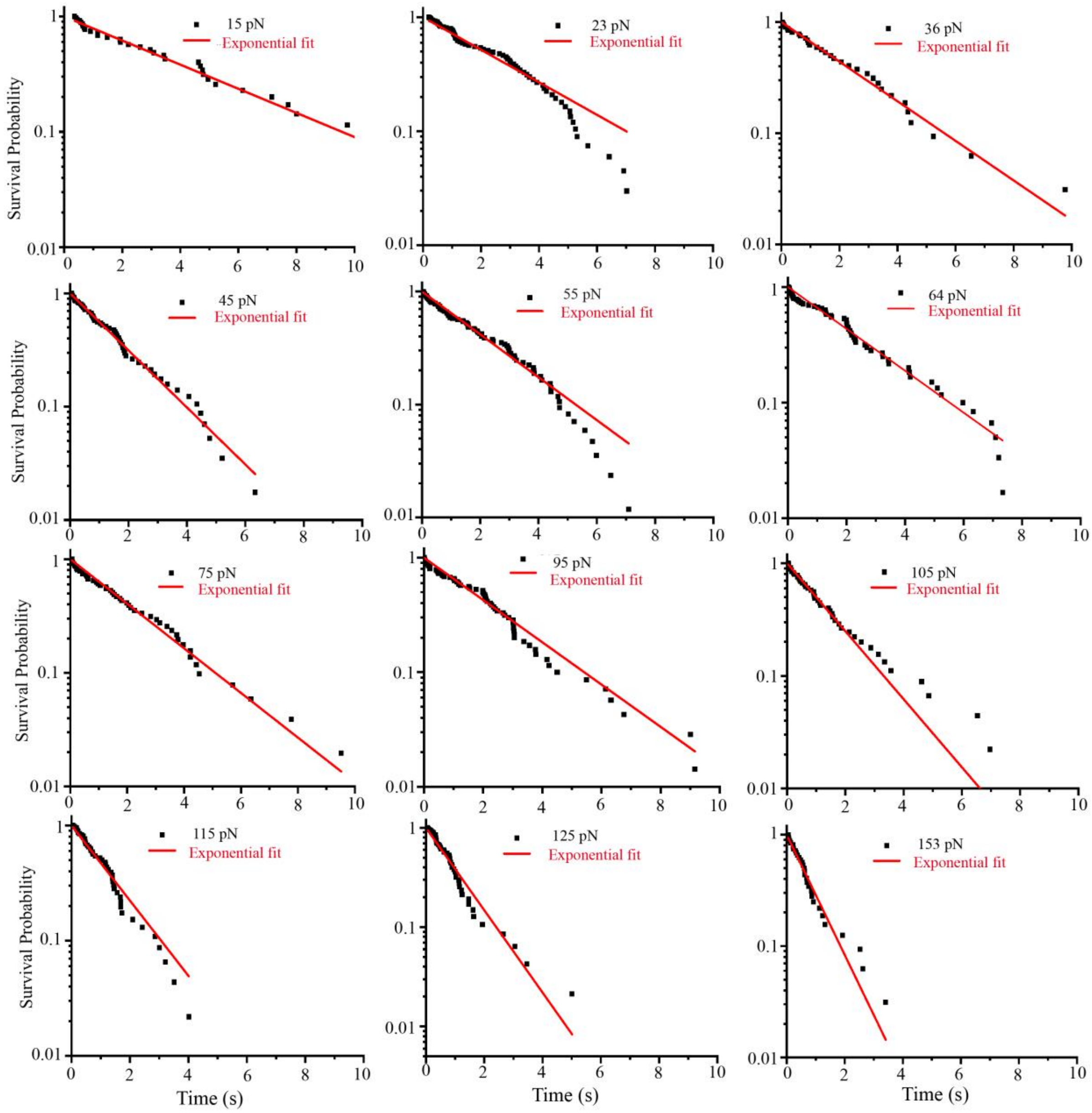


**Supplementary Figure 13: Exponential decay fitting of survival plot for *individual* tip-links comprising Cdh23 EC1-10-Pcdh15 EC1-2 at different clamping forces (in support of figure 3).** For Cdh23 EC1-10-Pcdh15 EC1-2, mono-exponential decay fitting of the survival plot is shown separately at each clamping force. The survival probabilities were derived from n = 35, 67, 32, 57, 86, 60, 51, 70, 45, 46, 47 and 32 data points for the clamping forces of 16, 23, 36, 45, 55, 64, 75, 95, 105, 115, 125, and 153 pN, respectively, from two independent experiments.

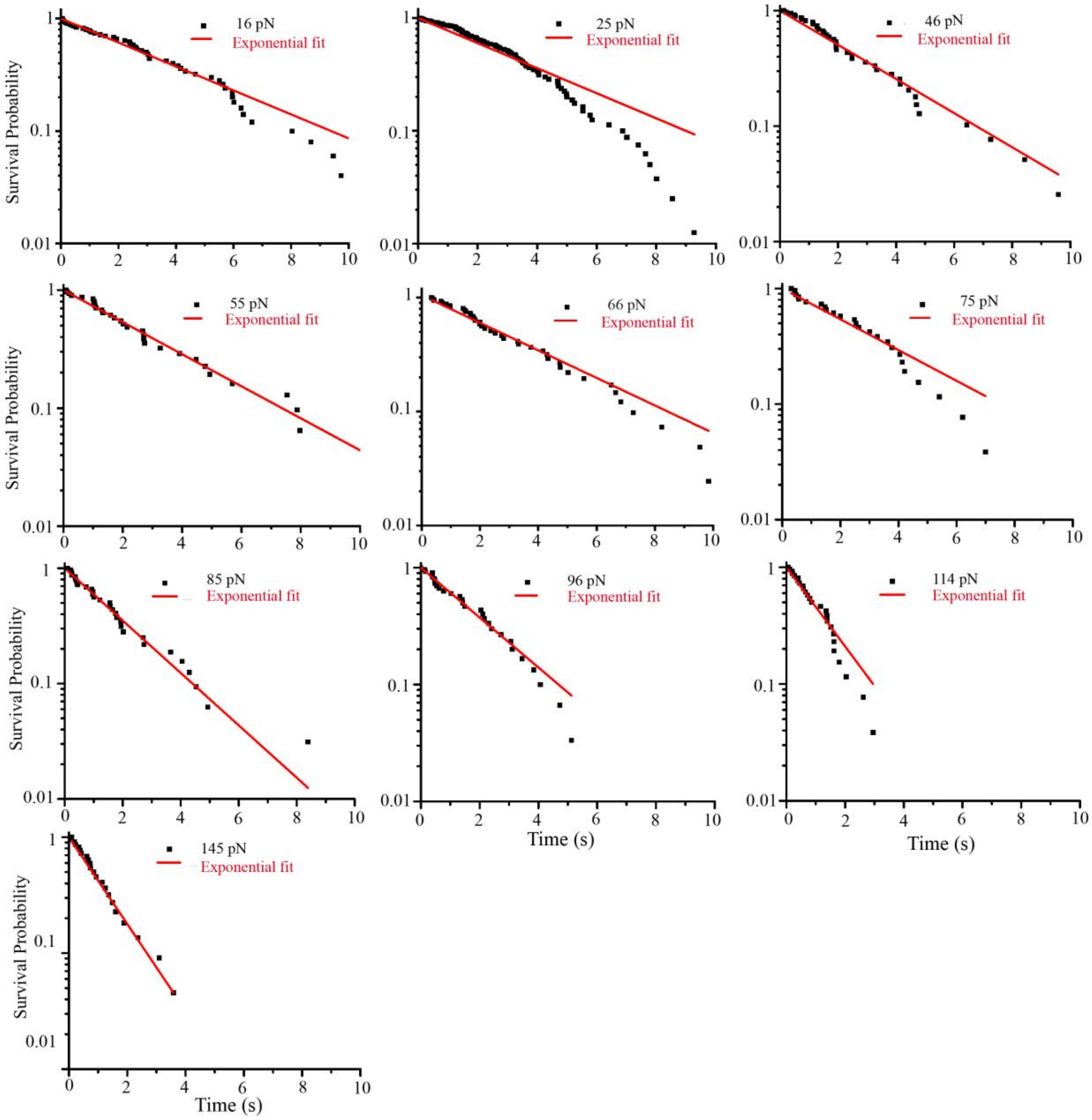


**Supplementary Figure 14: Exponential decay fitting of survival plot for *individual* tip-links comprising Cdh23 EC1-21-Pcdh15 EC1-2 at different clamping forces (in support of figure 3).** For Cdh23 EC1-21-Pcdh15 EC1-2, mono-exponential decay fitting of the survival plot is shown separately at each clamping force. The survival probabilities were derived from n = 49, 80, 39, 31, 41, 26, 32, 30, 26, and 22 data points for the clamping forces of 16, 25, 46, 55, 66, 75, 85, 96, 114, and 145 pN, respectively, from two independent experiments.

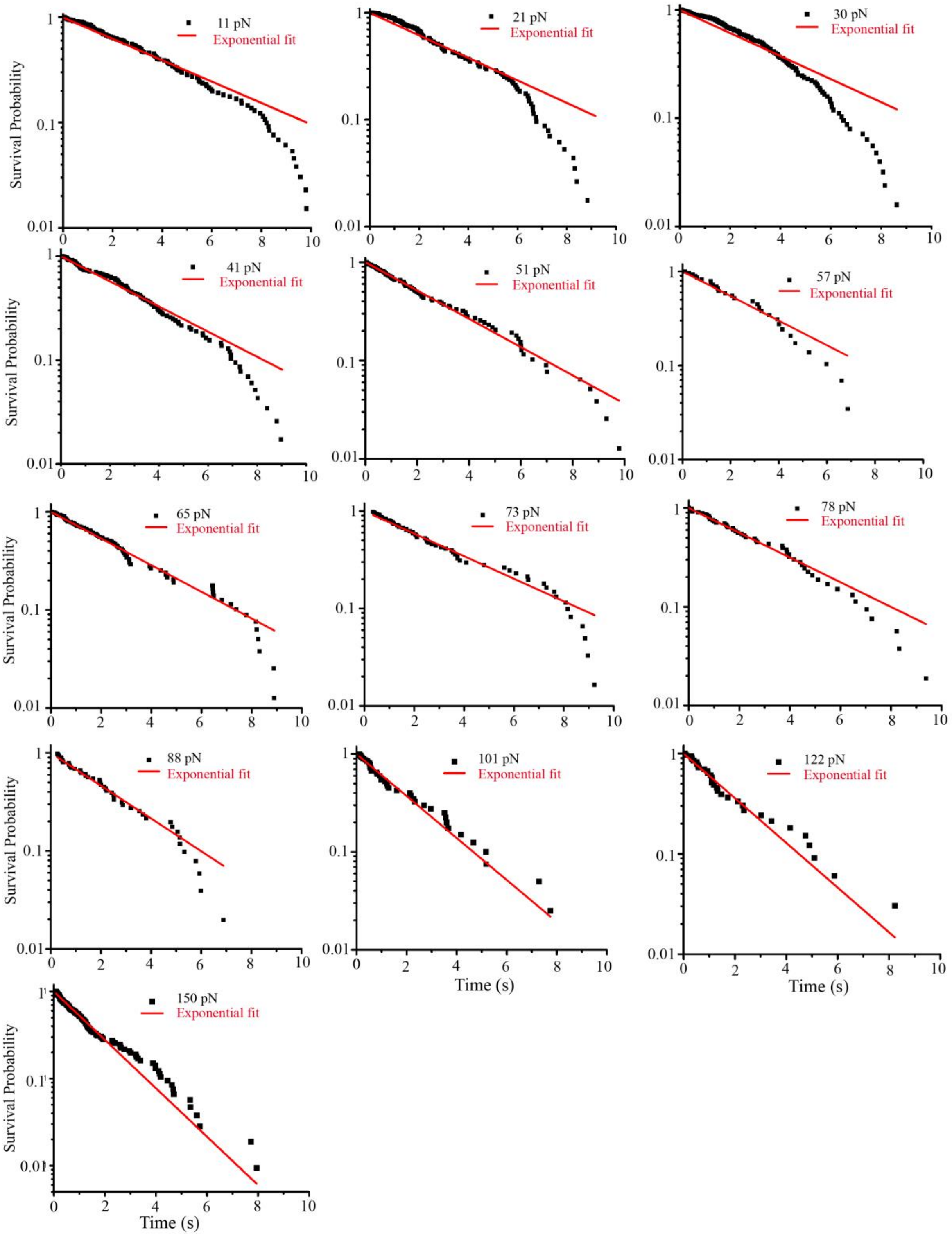


**Supplementary Figure 15: Exponential decay fitting of survival plot for *individual* tip-links comprising Cdh23 EC1-27-Pcdh15 EC1-2 at different clamping forces (in support of figure 3).** For Cdh23 EC1-27-Pcdh15 EC1-2, mono-exponential decay fitting of the survival plot is shown separately at each clamping force. The survival probabilities were derived from n = 131, 114, 126, 116, 78, 29, 79, 61, 52, 51, 40, 33, and 106 data points for the clamping forces of 11, 21, 30, 41, 51, 57, 65, 73, 78, 88, 101, 122, and 150 pN, respectively, from two independent experiments.

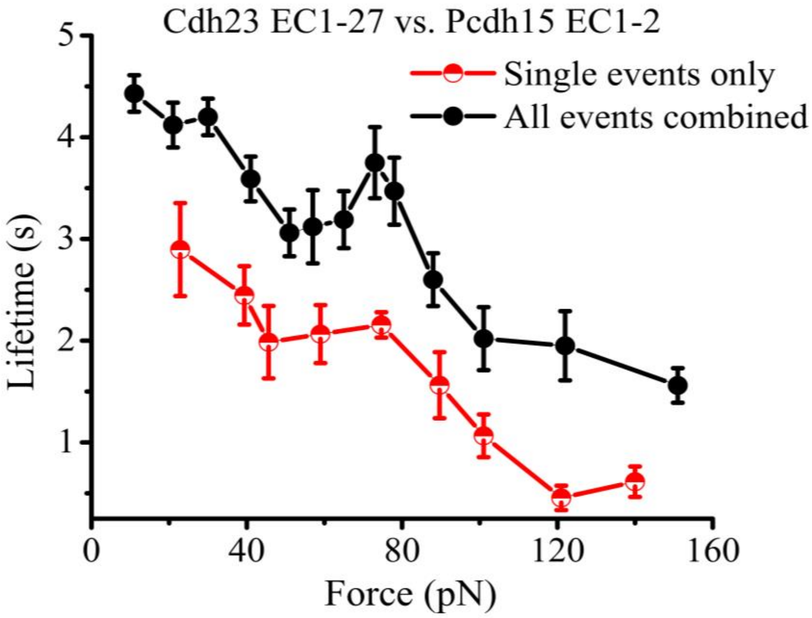


**Supplementary Figure 16. Unfolding prior to unbinding prolongs the bond lifetime (in support of figure 3).** Unbinding events without undergoing any unfolding registered a lower overall lifetime (red) compared to the events which showed unfolding prior to unbinding (black) for Cdh23 EC1-27-Pcdh15 EC1-2 interface. n=1016 for all events combined and 391 for single events. Data are presented as survival probability fit values ± fitting error.

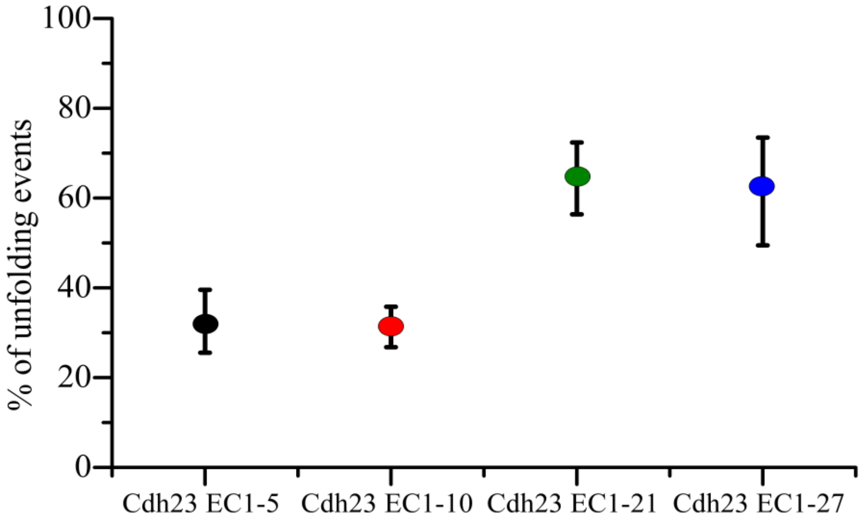


**Supplementary Figure 17. Percentage of unfolding events obtained in the force-clamp measurements for *individual* tip-links variants. (In support of figure 3).** Percentage of force-clamp events undergoing unfolding before unbinding increases with increasing the EC domain numbers. The data are presented as mean ± SEM from three independent experiments.

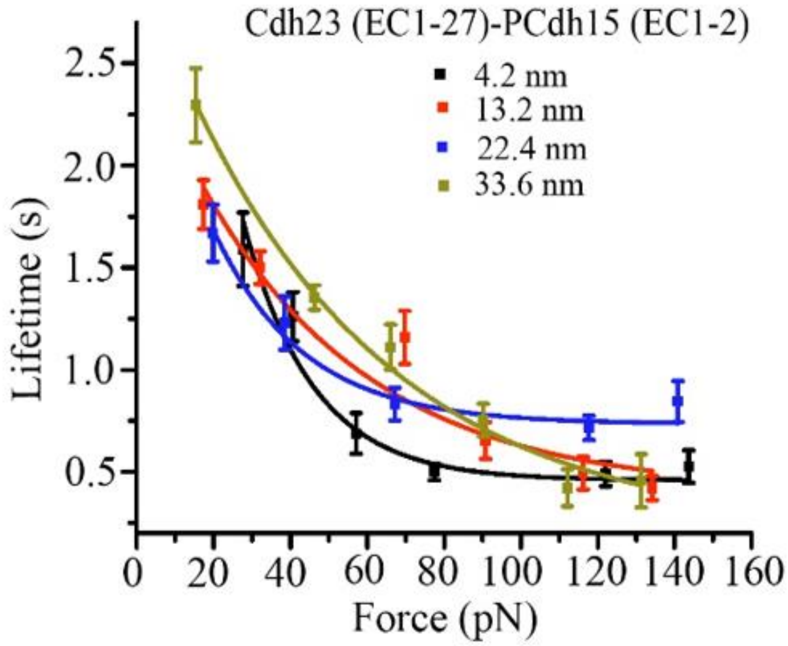


**Supplementary Figure 18: Force-lifetime data for all four major extensions for *individual* tip-links comprising Cdh23 EC1-27-Pcdh15 EC1-2 clamp measurements (in support of figure 3).** We fitted the force-lifetime data for all four extensions with the Bell's model to obtain the kinetic parameters like unfolding rate at zero force ($k_u^0$) and distance to transition state ($x_\beta$). These parameters we used to incorporate the unfoldings in the kinetic model fit. The total number of data points at each extension are obtained from step height distribution data for Cdh23 EC1-27-Pcdh15 EC1-2 (Fig 3e). Errors are the standard errors obtained from the exponential fitting of the survival probability curves.

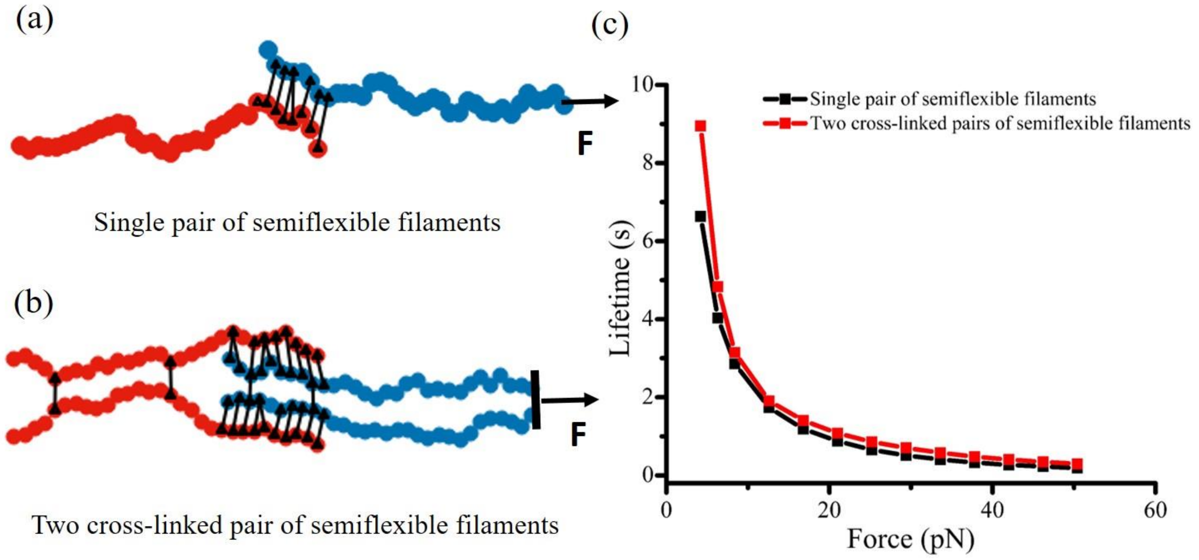


**Supplementary Figure 19: Force-dependent lifetime behaviour of semiflexible filaments linked with only slip-bonds (in support of figure 4). (a and b)** Schematic representation of single and double pair of semiflexible filaments coupled with multiple elastic slip-bonds. **(c)** The slip-bonded arrangement between chains resulted in exclusive slip-bond with force for both systems (n = 100 simulations per data point).

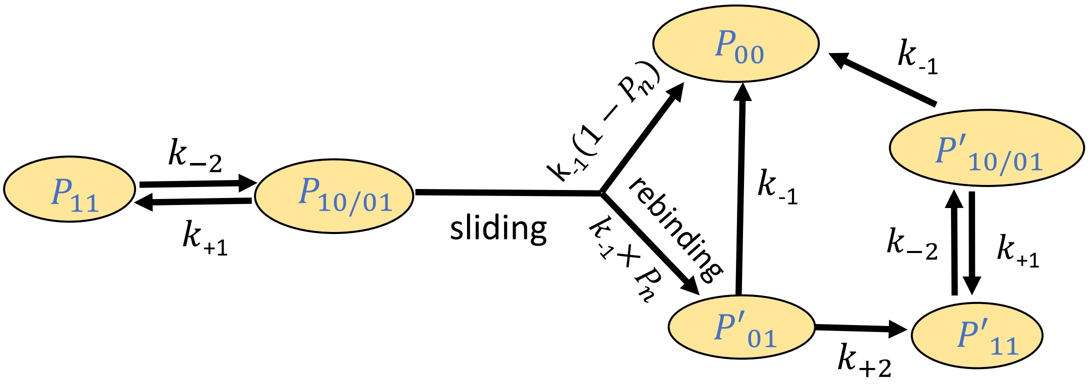


**Supplementary Figure 20: Dissociation paths for the wild-type tip-link interface.** Dissociation of the wild-type tip-link binding interface may occur through two competing pathways. One involves the conventional complete dissociation to $P_{00}$ while other involves the formation of force-induced stronger binding interface, $P'_{11}$, which is followed by the complete dissociation to $P_{00}$.

**Supplementary Table 1. One-sided F-statistical analysis for heterotetramer tip-link *complex***

| Force (pN) | Number of data points | SSR1 (Exp1) | SSR2 (Exp2) | df1 | df2 | (SSR1-SSR2)/(df1-df2) | SSR2/df2 | F-value | p |
|---|---|---|---|---|---|---|---|---|---|
| 12 | 86 | 0.0149 | N/A | N/A | N/A | N/A | N/A | N/A | N/A |
| 18 | 73 | 0.0388 | N/A | N/A | N/A | N/A | N/A | N/A | N/A |
| 31 | 65 | 0.0952 | N/A | N/A | N/A | N/A | N/A | N/A | N/A |
| 37 | 62 | 0.0488 | 0.0418 | 61 | 59 | 0.0035 | 0.00071 | 4.99 | 0.01 |
| 44 | 61 | 0.0392 | 0.0250 | 60 | 58 | 0.0071 | 0.00043 | 16.45 | 0 |
| 53 | 63 | 0.0601 | 0.0497 | 62 | 60 | 0.0052 | 0.00083 | 6.28 | 0.0033 |
| 72 | 60 | 0.2446 | 0.0271 | 59 | 57 | 0.1087 | 0.00048 | 228.49 | 0 |
| 85 | 60 | 0.0854 | 0.0469 | 59 | 57 | 0.0193 | 0.00082 | 23.45 | 0 |
| 107 | 60 | 0.2636 | 0.1160 | 59 | 57 | 0.0738 | 0.00204 | 36.18 | 0 |
| 133 | 111 | 0.3076 | 0.0515 | 110 | 108 | 0.1280 | 0.00048 | 268.33 | 0 |

SSR1 = Residual sum of squares for Single-exponential decay

SSR2 = Residual sum of squares for Double-exponential decay

df1 = Degree of freedom for Single-exponential decay

df2 = Degree of freedom for Double-exponential decay

N/A = Not available as we couldn't fit to the bi-exponential decay model

$$F = \frac{(SSR1 - SSR2)/(df1 - df2)}{SSR2/df2}$$

From the exponential decay fitting of the survival plots for the tetramer, we observed that for lower forces of 12, 18, and 31 pN we could explicitly fit the data to single-exponential decay so we couldn't estimate the F-values for these forces. This single-exponential fitting reflects the entire rebinding of the tip-link *complex* at lower forces. However, at intermediate forces of 37, 44, and 53 pN from the F-value statistics and p-value comparison (**confidence level of**

**0.01**), we observed that double-exponential fit is also possible but it didn't improve the fitting further. Also, we couldn't detect the double-exponential behavior in the survival plots. The lower lifetime decay component with very low probability is corresponds to the fraction of tip-link *complex* that didn't undergo rebinding. The emergence of this bi-exponential behavior was profoundly observed at higher forces of 72, 85, 107, and 133 pN since the probability of rebinding will decrease even further at higher forces. We also noticed this behavior from the corresponding amplitudes at higher forces as shown in **Supplementary Figure 4**.
