# Supplementary material for: Emergence of slip-ideal-slip behavior in tip-links serve as force filters of sound in hearing
Source: Nat Commun. 2024 Feb 21;15:1595. doi: 10.1038/s41467-024-45423-8 (PMC10881517; doi:10.1038/s41467-024-45423-8)
Supplement: Supplementary file 2 — Description of additional supplementary files [file 41467_2024_45423_MOESM2_ESM.pdf]

Description of Additional Supplementary Files

Supplementary Movie 1

The dimeric arrangement of two semiflexible filaments, partially attached to one another via elastic bonds with catch-slip dissociation characteristics. The bonds dissociate in the presence of load when one of the filaments is pulled externally.

Supplementary Movie 2

The tetrameric arrangement, where the two filaments from each of the dimeric arrangements are pulled simultaneously with the external force at one end. Additionally, the anchored filaments are cross-linked resembling the cis-dimers of Pcdh15 (red). We also cross-linked the polymer couples that are pulled, at their cross-linking interface. This polymer couple resembles Cdh23 (blue). We observed the rebinding of the bonds in this tetrameric arrangement before the complete unbinding.
