# Supplementary material for: Emergence of slip-ideal-slip behavior in tip-links serve as force filters of sound in hearing
Source: Nat Commun. 2024 Feb 21;15:1595. doi: 10.1038/s41467-024-45423-8 (PMC10881517; doi:10.1038/s41467-024-45423-8)
Supplement: Supplementary file 5 — Reporting Summary [file 41467_2024_45423_MOESM5_ESM.pdf]

Corresponding author(s): Sabyasachi Rakshit
Last updated by author(s): Dec 7, 2023

Reporting Summary

Nature Portfolio wishes to improve the reproducibility of the work that we publish. This form provides structure for consistency and transparency in reporting. For further information on Nature Portfolio policies, see our Editorial Policies and the Editorial Policy Checklist.

Statistics

For all statistical analyses, confirm that the following items are present in the figure legend, table legend, main text, or Methods section.

n/a	Confirmed	
☐	☒	The exact sample size (*n*) for each experimental group/condition, given as a discrete number and unit of measurement
☐	☒	A statement on whether measurements were taken from distinct samples or whether the same sample was measured repeatedly
☐	☒	The statistical test(s) used AND whether they are one- or two-sided<br>*Only common tests should be described solely by name; describe more complex techniques in the Methods section.*
☒	☐	A description of all covariates tested
☒	☐	A description of any assumptions or corrections, such as tests of normality and adjustment for multiple comparisons
☐	☒	A full description of the statistical parameters including central tendency (e.g. means) or other basic estimates (e.g. regression coefficient) AND variation (e.g. standard deviation) or associated estimates of uncertainty (e.g. confidence intervals)
☐	☒	For null hypothesis testing, the test statistic (e.g. *F*, *t*, *r*) with confidence intervals, effect sizes, degrees of freedom and *P* value noted<br>*Give P values as exact values whenever suitable.*
☒	☐	For Bayesian analysis, information on the choice of priors and Markov chain Monte Carlo settings
☒	☐	For hierarchical and complex designs, identification of the appropriate level for tests and full reporting of outcomes
☒	☐	Estimates of effect sizes (e.g. Cohen's *d*, Pearson's *r*), indicating how they were calculated

Our web collection on statistics for biologists contains articles on many of the points above.

Software and code

Policy information about availability of computer code

Data collection	AFM data has been collected using JPK SPM desktop software provided by JPK. All MD simulations were run using GROMACS 2020.4 patched with PLUMED 2.7.4.
Data analysis	Recorded force curves were analyzed in home-written codes in MATLAB R2020a for lifetime and step-height measurements. Exponential decay fitting of the survival plots was performed using Origin 2018. SMD simulation data was analyzed using PLUMED, VMD, MDAnalysis, PyInteraph, and gromacs-fda for computing various parameters.

For manuscripts utilizing custom algorithms or software that are central to the research but not yet described in published literature, software must be made available to editors and reviewers. We strongly encourage code deposition in a community repository (e.g. GitHub). See the Nature Portfolio guidelines for submitting code & software for further information.

Data

Policy information about availability of data

All manuscripts must include a data availability statement. This statement should provide the following information, where applicable:

- Accession codes, unique identifiers, or web links for publicly available datasets
- A description of any restrictions on data availability
- For clinical datasets or third party data, please ensure that the statement adheres to our policy

All the data-sets are provided in the main manuscript, supplementary file and source data file. MATLAB codes are available in github repository. Other data is available upon reasonable request to authors.

Research involving human participants, their data, or biological material

Policy information about studies with human participants or human data. See also policy information about sex, gender (identity/presentation), and sexual orientation and race, ethnicity and racism.

Reporting on sex and gender	NA
Reporting on race, ethnicity, or other socially relevant groupings	NA
Population characteristics	NA
Recruitment	NA
Ethics oversight	NA

Note that full information on the approval of the study protocol must also be provided in the manuscript.

Field-specific reporting

Please select the one below that is the best fit for your research. If you are not sure, read the appropriate sections before making your selection.

☒ Life sciences ☐ Behavioural & social sciences ☐ Ecological, evolutionary & environmental sciences

For a reference copy of the document with all sections, see nature.com/documents/nr-reporting-summary-flat.pdf

Life sciences study design

All studies must disclose on these points even when the disclosure is negative.

Sample size	Sample-size calculation was not performed, however, the sizes were sufficiently large to reduce the errors in the estimation of the lifetimes from the survival plots from force-spectroscopy studies.
Data exclusions	We have excluded the force-curves from the analysis for which the lifetimes are too small, i.e. less than 0.01 sec, based on the temporal resolution of our AFM instrument.
Replication	All the reported experiments and simulations were repeated in triplicates. For every independent experiment, a new set of cantilever and protein batch was used.
Randomization	No randomization was needed.
Blinding	Blinding was not required as we have considered all the data for analysis. The analyses were done using already standardized protocols and common softwares.

Reporting for specific materials, systems and methods

We require information from authors about some types of materials, experimental systems and methods used in many studies. Here, indicate whether each material, system or method listed is relevant to your study. If you are not sure if a list item applies to your research, read the appropriate section before selecting a response.

Materials & experimental systems

n/a	Involved in the study
☒	☐ Antibodies
☐	☒ Eukaryotic cell lines
☒	☐ Palaeontology and archaeology
☒	☐ Animals and other organisms
☒	☐ Clinical data
☒	☐ Dual use research of concern
☒	☐ Plants

Methods

n/a	Involved in the study
☒	☐ ChIP-seq
☒	☐ Flow cytometry
☒	☐ MRI-based neuroimaging

Eukaryotic cell lines

Policy information about cell lines and Sex and Gender in Research

Cell line source(s)	Thermo Fisher Scientific - Expi-CHO cell lines
Authentication	No authentication was performed.
Mycoplasma contamination	Tested negative by the supplier and no Mycoplasma contamination is noticed.
Commonly misidentified lines (See ICLAC register)	No commonly misidentified lines.
